# Revealing the charge transport physics in metallic coordination nanosheets by thermoelectric and magnetotransport measurements

**DOI:** 10.1126/sciadv.adt9196

**Published:** 2025-04-09

**Authors:** Tian Wu, Xinglong Ren, David Cornil, Claudio Quarti, Ian E. Jacobs, Lu Zhang, Naoya Fukui, David Beljonne, Hiroshi Nishihara, Henning Sirringhaus

**Affiliations:** ^1^ Cavendish Laboratory, University of Cambridge, Cambridge, UK.; ^2^ Laboratory for Chemistry of Novel Materials, University of Mons, Mons, Belgium.; ^3^ Research Institute for Science and Technology, Tokyo University of Science, Tokyo, Japan.

## Abstract

We have studied the charge transport physics of high-quality conducting coordination nanosheets films based on the benchmark material copper benzenehexathiol (CuBHT) by measuring multiple thermoelectric and magnetotransport coefficients on the same film. The films exhibit a metallic temperature dependence of the conductivity over a wide temperature range, but below 15 kelvin charge transport becomes dominated by weak localization and electron-electron interactions. Temperature-dependent Hall, Seebeck, and Nernst measurements consistently indicate the existence of ambipolar transport characteristics in CuBHT. A two-band analysis has been used to extract transport parameters for electron and hole carriers as a function of temperature. The results show that contributions from electron and hole conduction in CuBHT are of comparable magnitude, revealing the complexity of charge transport and allowing one to identify strategies for enhancing the thermoelectric transport coefficients of such conducting coordination nanosheets.

## Teaser

Charge transport in conducting coordination nanosheets is governed by contributions from multiple electron and hole bands.

## INTRODUCTION

Since the groundbreaking discovery of graphene (*1*, *2*), many types of two-dimensional (2D) materials with extended π-conjugation similar to graphene have attracted great interest (*3*–*5*). Among them, coordination nanosheets (CONASHs) have garnered considerable attention due to their chemically designable nature and unique structural/electrical properties. In particular, CONASHs based on π-conjugated ligands have shown tremendous potential in the field of charge transport (*4*, *6*, *7*). The remarkable electronic properties of π-conjugated CONASHs arise from their 2D structure, which consists of transition metal ions coordinated with ligand molecules, resulting in π-d conjugation and electron delocalization across the 2D layers (*8*). This arrangement gives rise to a fascinating interplay between molecular-level interactions and macroscopic charge transport properties. The ability to manipulate and understand charge transport within these CONASHs holds great promise for the development of electronic devices such as transistors, sensors, and energy storage systems (*9*, *10*).

The multidentate ligand benzenehexathiol (BHT) has attracted particular attention. In 2013, a conducting CONASH composed of a nickel-BHT complex (NiBHT) was reported by Kambe *et al.* (*11*, *12*), which exhibited oxidation-tunable conductivity with highest conductivity reached in a fully oxidized state. The experimental findings have sparked theoretical interest in BHT-based coordination polymers, leading to predictions of organic topological insulators and half-metallic spin lattices (*13*, *14*). In addition to nickel, copper ions have also been extensively studied, ranging from bisdithiolene complexes to 1D coordination polymers and 2D networks (*15*–*17*). In 2015, Huang *et al.* (*17*) first reported a CONASH based on CuBHT, which exhibited a high conductivity ~1580 S/cm. Both the monolayer and the bulk phase of CuBHT have been theoretically predicted to exhibit superconductivity (*18*). In follow-on papers, Huang *et al.* (*19*, *20*) also reported a modified synthesis process, which led to a CuBHT film with even higher conductivity and emergence of superconductivity at ~0.25 K. The metallic nature of CuBHT was confirmed by temperature-dependent conductivity (*19*) and further studied by ultrafast terahertz spectroscopy (*21*). However, for this benchmark material, inconsistent charge transport properties have been reported by different groups, which implies that the charge transport physics of CuBHT remains insufficiently well understood. For example, both negative (*22*) and positive (*23*) Seebeck coefficients can be found in the literature. Moreover, publications report variable conductivity values and decoupling the contributions from charge density and carrier mobility to the observed metallic conductivity remains challenging. Conventional field-effect transistor measurements to extract mobilities are not suitable for samples with metallic conductivities due to the high bulk carrier density resulting in negligible field-effect modulation. Hall effect measurements, which provide a potential means to determine charge carrier concentration, have not been reported in CuBHT yet. Consequently, to better understand the mechanisms behind the high conductivity and gain more detailed insight into the electronic structure and the transport physics, a detailed and systematic study of the charge transport properties of this important CONASH model system is urgently needed.

Here, we report such a detailed study of the temperature-dependent transport properties of CuBHT, including magnetoresistance (MR), Hall, Seebeck, and Nernst measurements based on a multifunctional device architecture that allows for measurements of these different transport coefficients on the same device. Our fabricated CuBHT devices exhibit high conductivities over 1500 S/cm at low temperatures with a metallic temperature dependence, similar to what have been previously reported (*19*). From our temperature- and magnetic field–dependent transport study, we observe clear evidence for a multiband, ambipolar charge transport regime in CuBHT, in which both electron and hole carriers contribute to the conduction. By measuring multiple transport coefficients on the same device, we aim to differentiate between their contributions and extract mobilities and concentrations of electrons and holes separately. We find that both electrons and holes have mobilities on the order of ~5 cm^2^ V^−1^ s^−1^ and carrier densities of ~10^21^ cm^−3^ and that they contribute almost equally to conduction over a wide temperature range, which provides an explanation for the inconsistent observations in the literature. Our study shows that, by combining magnetotransport and thermoelectric characterizations, it is possible to gain deeper insight into the charge transport properties of CuBHT. The method may also be applicable to other organic and hybrid material systems with similar ambipolar charge transport characteristics.

## RESULTS

### Crystal structure and chemical analysis

**Fig. 1.**
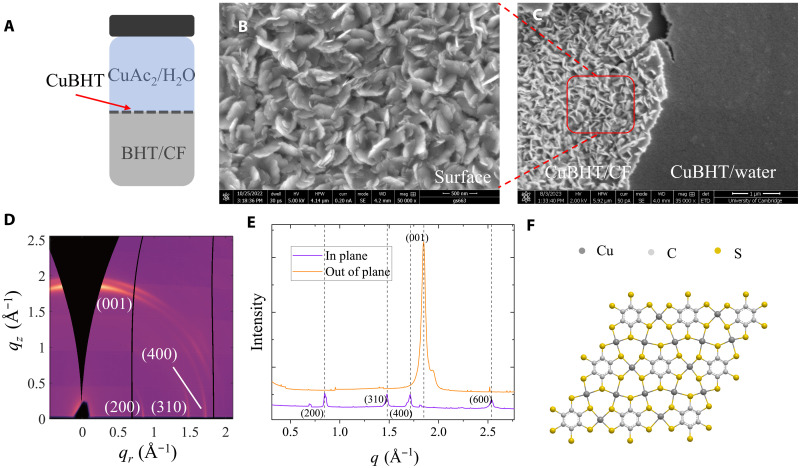
Microstructural characterization of CuBHT films used in this work. (**A**) Illustration of the liquid-liquid interfacial synthesis process. (**B**) SEM image of the surface of a CuBHT film picked up from the liquid-liquid interface with a Si substrate; the surface of the film was in contact with the organic phase during the growth. Scale bar, 500 nm. (**C**) SEM image of film region that has the CuBHT/CF side exposed (left) and the CuBHT/water side (right). Scale bar, 1 μm. This film was scratched by a needle and, after scratching some part of the film, delaminated and folded back on itself, exposing the original bottom surface of the film on the Si substrate. (**D**) GIWAXS pattern of a CuBHT film. (**E**) 1D integrated plot of in-plane and out-of-plane GIWAXS of CuBHT. (**F**) Schematic of the chemical structure of CuBHT.

CuBHT films were prepared based on a widely reported liquid-liquid interfacial method between an organic, chloroform (CF)–based phase containing the BHT and an aqueous phase with the copper precursor (Fig. 1A) (*19*). Some modifications were made to the reported recipes, and the detailed process is described in Materials and Methods. The scanning electron microscopy (SEM) image (Fig. 1B) shows that CuBHT films formed on the CF side consist of 2D platelets, which is consistent with its 2D nature. These platelet crystals have a lateral size of ~100 nm, which is consistent with atomic force microscopy (AFM) images (fig. S1A) and, in the SEM images, appears with a somewhat random orientation. A 45° cross-sectional image was also taken to get a side view of the CuBHT film (fig. S1B). Toward the other side of the film, which was in contact with the water phase, the films become smoother and more compact, implying that the CuBHT crystals are stacked more densely at the water side. This can be directly observed in Fig. 1C, which shows a portion of the film flipped upside down by scratching, so that both the sides of the film can be compared in the same image area. This morphological difference is consistent with previously reported results (*17*). θ-2θ x-ray diffraction (XRD) patterns (fig. S2) of our CuBHT films exhibit a strong peak at 2θ ~ 25.9°, which corresponds to a lattice spacing of 3.43 Å. This spacing value is close to the reported interlayer distance of CuBHT (001) planes (*19*, *23*). In addition, grazing incidence wide-angle x-ray scattering (GIWAXS) was performed to confirm the crystalline packing in our CuBHT films (Fig. 1D). In the out-of-plane direction, a strong (001) peak at *q* ~ 1.84 Å^−1^ was observed, which corresponds to a lattice spacing of 3.40 Å, consistent with the XRD data. In the in-plane direction, a series of GIWAXS peaks can be indexed with (*hk*0) (Fig. 1E), which match quite well with those reported for CuBHT in the literature (*19*). For the indexing of the diffraction peaks in Fig. 1 (D and E), we therefore used the crystal structure reported in ref. (*19*). The (001) peak is stronger in the out-of-plane direction, whereas the family of (*h*00) peaks show a higher intensity in the in-plane direction, which indicates the dominance of face-on textures in CuBHT films. From the SEM image, there is a dense layer close to side of the film that was in contact with the water phase, whereas the surface in contact with the organic phase only consists of apparently randomly stacked platelets, which do not have a clear preferential orientation. A plausible interpretation is that the dense layer is more crystalline with the face-on structure and contributes mostly to the GIWAXS pattern, whereas the randomly stacked platelets are less crystalline as a film with randomly oriented crystallites should lead to a more isotropic, ring-like diffraction pattern in GIWAXS.

Chemical analysis of our CuBHT films is performed with energy-dispersive x-ray spectrometry (EDS) and x-ray photoelectron spectroscopy (XPS). The EDS analysis (fig. S3) shows a weight percentage of Cu:S at around 1:1.2, corresponding to an atomic percentage around 1:2. This agrees well with the expected CuBHT chemical composition of [Cu_3_(C_6_S_6_)]*_n_* (*19*, *23*), which is shown in Fig. 1F. The XPS results (fig. S4) show clear peaks from Cu 2p and S 2p. The Cu 2p peaks did not show obvious satellite peaks, which is a clear indication of the Cu^1+^ state and the absence of Cu^2+^ state (*17*, *23*). The metallic character of CuBHT is revealed by ultraviolet photoelectron spectroscopy (UPS), which shows a nonzero density of states (DOS) at the Fermi level (fig. S4). In summary, our CuBHT films have a chemical composition and thin film microstructure that resembles those found in previous reports, and in the following sections, we will show that our CuBHT films also have very good charge transport properties with high electrical conductivity, allowing for a reliable transport study on materials that are representative of literature studies.

### Charge transport properties

**Fig. 2.**
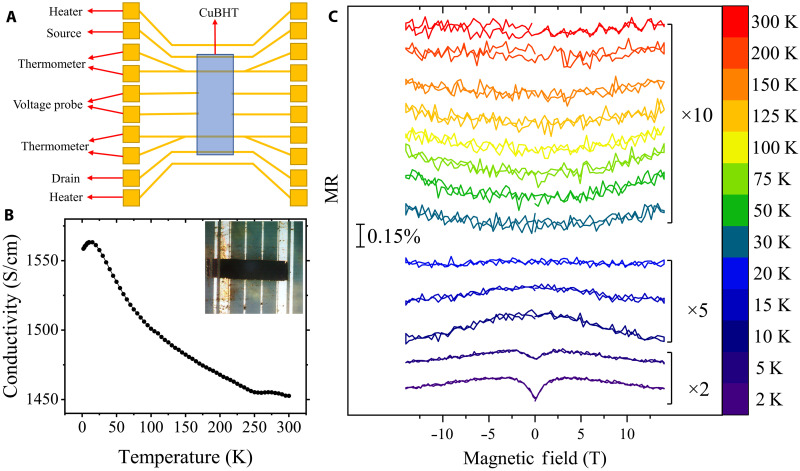
Measurements of temperature-dependent conductivity and MR in CuBHT. (**A**) Device structure used in this work. (**B**) Temperature-dependent conductivity of CuBHT. Inset: Optical microscopy image of the device. (**C**) MR of CuBHT from −14 to 14 T at different temperatures. The MR curves at different temperatures are vertically shifted and magnified with different factors so that they can be displayed in the same figure. The scale bar is 0.15%.

The electrical conductivity (σ) of CuBHT was investigated using a multifunctional Hall bar device as shown in Fig. 2A. The film was mechanically patterned to a dimension of 420 μm in length (*L*) and ~160 μm in width (*W*) using a micropositioner with a tungsten needle under an optical microscope. The thickness (*t*) of film was measured to be 200 to 300 nm by a Dektak profilometer, which was also confirmed with AFM measurements (fig. S5). The thickness varies somewhat from sample to sample as the reaction conditions in the interfacial synthesis are difficult to control precisely. The roughness of the top surface is ~19.6 nm (fig. S5), which indicates that the film is relatively smooth considering its thickness, further confirming the desirable uniformity of the CuBHT film and the reliability of the calculated σ value.

The temperature-conductivity relationship is shown in Fig. 2B, with the optical image of a real device shown in the inset. σ gradually increases with decreasing temperature from a room temperature value of 1452 S/cm, which means that our CuBHT films are metallic over a wide range of temperature, consistent with UPS results. However, below around 15 K, the conductivity starts to decrease with decreasing temperature. This unusual decrease indicates that a different transport mechanism dominates charge transport in CuBHT at the lowest temperatures; this will be discussed next.

Several mechanisms can lead to positive dσ/d*T* at low temperatures, including weak localization (WL), electron-electron interaction (EEI), and the Kondo effect. To figure out which mechanism dominates at *T* < 15 K, we performed MR measurements on the same device shown in Fig. 2B because those mechanisms behave differently under a magnetic field (*B*). Temperature-dependent MR measurements in a perpendicular magnetic field are presented in Fig. 2C, with MR defined as $\mathrm{MR}=\frac{R\left(B\right)-R_{0}}{R_{0}}\times 100\%$, where *R*(*B*) is the resistance at a certain magnetic field and *R*_0_ is the resistance at zero field. At 30 K and above, the MR is positive and varies approximately parabolically with *B*, which can be well explained by the classical MR effect commonly observed in metals due to the Lorentz force. In general, the magnitude of positive MR is very small (<0.02%), implying that the average carrier mobility (μ) is on the order of 1 to 10 cm^2^ V^−1^ s^−1^ [estimated from MR ~ (μ*B*)^2^ (*24*, *25*)]. At 20 K, a negative component starts to appear at low fields, and it dominates over the full range of magnetic field at 15 and 10 K.Consistently, the conductivity also starts to decrease with decreasing temperature over this temperature range. Angular-dependent MR measurements confirm that this negative component is highly anisotropic and becomes negligible when the direction of the magnetic field is in-plane (fig. S6). The observation of such anisotropic, negative MR indicates that 2D WL is the main origin of the positive dσ/d*T* at low *T*: For EEI, the MR is expected to be isotropic and positive; for the Kondo effect, the MR should be isotropic and negative. Although the thickness of the CuBHT films is several hundred nanometers, its quasi-2D nature of charge delocalization is not very unexpected considering the anisotropic packing structure revealed by GIWAXS. We also found that the phase coherence length extracted from 2D WL theory is around tens of nanometers and increases with decreasing temperature (table S1), similar to values reported in some conducting polymers (*26*). However, 2D WL is not the only origin of decreased conductivity at low *T*. At 5 and 2 K, the MR becomes positive at low fields and negative at high fields, and the positive component is almost isotropic (see fig. S6D), which may be attributed to EEI. Overall, the MR results suggest that the unusual decrease in conductivity at low *T* originates from a combination of 2D WL and EEI, with the latter one becoming stronger as *T* approaches 2 K.

**Fig. 3.**
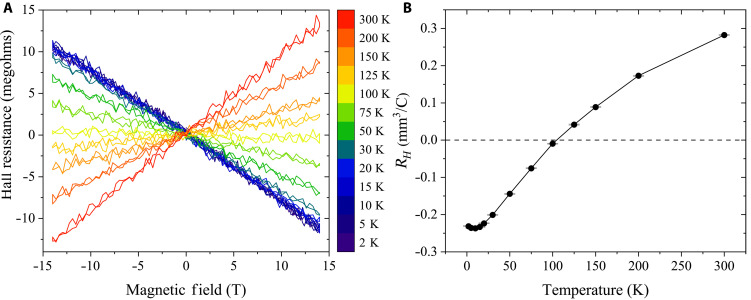
Measurement of the temperature-dependent Hall effect in CuBHT. (**A**) Hall resistance versus magnetic field at different temperatures. (**B**) Calculated Hall coefficients from (A). The zero-field offset resistances are subtracted.

To get further insight into charge transport in CuBHT, temperature-dependent Hall measurements were performed simultaneously with MR measurements. The Hall resistance versus *B* curves are shown in Fig. 3A. This study shows the experimental observation of clear Hall signals in CuBHT, and such a good signal-to-noise ratio has not been achieved in other coordination polymers before. Thanks to the high-quality Hall effect data, Hall coefficients (*R_H_*) at different temperatures can be reliably extracted from the slope of the Hall resistance as a function of magnetic field (Fig. 3B). The most notable observation is that the Hall coefficient changes sign at around 100 K: At high temperatures, *R_H_* is positive and gradually decreases with decreasing temperature. Below 100 K, it turns negative and increases in magnitude when lowering the temperature further, suggesting a crossover from p-type to n-type dominated transport. In a Hall experiment, electrons and holes drift in opposite directions in response to the applied longitudinal electric field and are deflected toward the same side of the channel in the transverse direction by the Lorentz force they experience, i.e., the resulting Hall voltages arising from electrons and holes tend to have opposite signs. The observation of a sign reversal in the Hall coefficient indicates that charge transport in CuBHT is ambipolar, i.e., electron and hole carriers in different bands contribute to the conductivity. In such an ambipolar system, estimating charge density simply from $R_{H}=\frac{1}{n_{h/e}e}$ (*n_h/e_* is the hole/electron density, and *e* is elementary charge) is erroneous. For example, a near-zero *R_H_* around 100 K would lead to an unphysically large charge density. Instead, quantitative estimation of charge densities and mobilities in such ambipolar systems requires a more complicated two-carrier model taking into consideration the contributions from both electrons and holes, which will be discussed below.

**Fig. 4.**
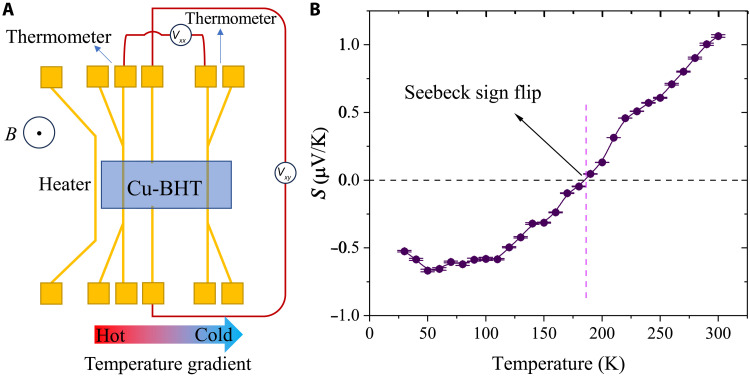
Measurement of the temperature-dependent Seebeck coefficient in CuBHT. (**A**) Device structure for thermoelectric measurements. (**B**) Temperature-dependent Seebeck coefficient of CuBHT.

The Seebeck coefficient (*S*), which is affected by the energy distribution of charge carriers in relation to the Fermi level (*E_F_*), offers crucial insight into the electronic structure along the charge carrier transport pathways. *S* is positive if holes are the majority carriers and negative if electrons are the majority carrier as both types of carriers preferentially diffuse in the same direction towards the cold end in a temperature gradient. Figure 4A shows a schematic of our thermoelectric measurement configuration, in which an on-chip heater generates an in-plane temperature gradient, and *S* is obtained by measuring the voltage difference between two electrodes that also serve as resistance thermometers (more details in figs. S7 to S9) (*23*, *27*, *28*). As shown in Fig. 4B, *S* is positive (p-type) at temperatures above 180 K but flips its sign to negative (n-type) at lower temperatures. This suggests that the majority carrier in CuBHT changes from holes to electrons when the temperature decreases. The magnitude of *S* in CuBHT is very low (<1 μV/K) and comparable to those of gold thin films (*29*), which suggests that the measured *S* should be treated as an upper bound (section S3) and the true magnitude might be smaller. Such a low magnitude of *S* usually indicates the coexistence of electrons and holes. We also emphasize that we observe a similar sign flip in *R_H_*. As stated above, the Hall and Seebeck measurements were performed on the same device. As shown in Figs. 3B and 4B, at *T* > 180 K, both *R_H_* and *S* are positive, which indicates that holes contribute more to charge transport, whereas at *T* < 110 K, both are negative, representing a higher contribution from electrons. At the range of 110 to 180 K, *R_H_* is positive whereas *S* is negative, and their different critical temperatures for sign flip can be explained by either the different functional forms of *R_H_* and *S* or by a small contribution to the measured thermal voltage from the Au electrodes. In general, our temperature-dependent Hall and Seebeck measurements provide clear evidence that there are two types of carriers contributing to charge transport in CuBHT and that electrons and holes are compensating each other, leading to generally small values of both *R_H_* and *S*.

Last, we also measured the Nernst effect, which is a transverse thermoelectric coefficient that can be used to probe the energy dependence of the scattering processes experienced by the mobile quasiparticles. Its relation to the Seebeck effect is analogous to that between the Hall effect and the longitudinal electrical conductivity. The Nernst coefficient (υ) is defined as the transverse voltage induced by a longitudinal temperature gradient and vertical magnetic field as shown in Fig. 4A (more details in fig. S11)

$$\upsilon =\frac{E_{y}}{B_{z}}\frac{1}{\nabla_{x}T}$$ (1)

where *E_y_* is the transverse electric field, *B_z_* is the vertical magnetic field, and ∇*_x_T* is the longitudinal temperature gradient. Substituting *E_y_ = V_xy_*/*W* and $\nabla_{x}T$ *= ∆T*/*L* (*V_xy_* is the transverse voltage), the equation can be written as

$$\upsilon =\frac{L}{W}\frac{V_{\mathrm{xy}}}{B_{z}}\frac{1}{∆T}$$ (2)

**Fig. 5.**
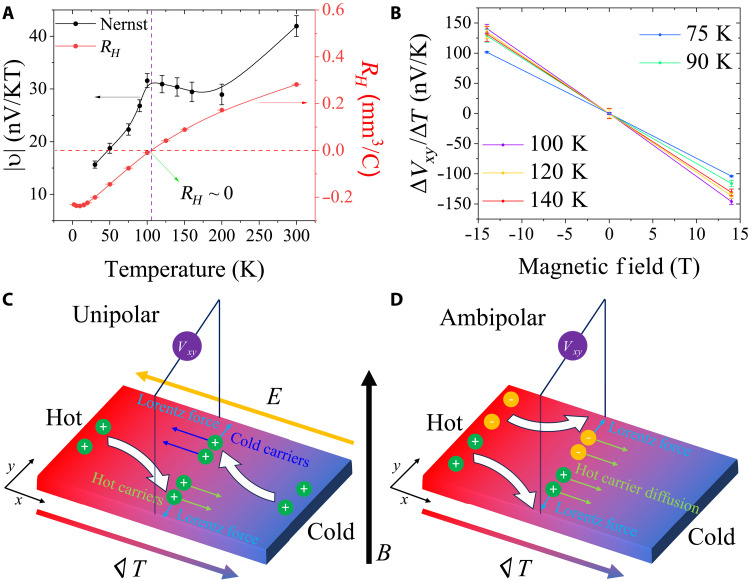
Measurement of the temperature-dependent Nernst coefficient in CuBHT, performed on the same device as shown in Figs. 3 and 4. (**A**) Temperature-dependent absolute Nernst coefficient value plotted with the Hall coefficient measured in Fig. 3B. (**B**) Transverse thermopower ∆*V_xy_*/∆*T* plotted against magnetic field at different temperatures. (**C**) Schematic of Nernst effect in a unipolar system: Contributions from hot carriers driven by the temperature gradient and cold carriers driven by the Seebeck electric field tend to cancel out each other. (**D**) Schematic of the Nernst effect in an ambipolar system, for which contributions from electrons and holes add up.

Results for temperature-dependent Nernst measurements are summarized in Fig. 5A with a detailed plot of ∆*V_xy_*/∆*T* versus magnetic field shown in Fig. 5B. Compared with the Seebeck effect, the Nernst effect is much less explored in most materials because υ is usually very small in a one-carrier system due to the so-called “Sondheimer cancellation.” This refers to the contributions to the transverse Nernst voltage from hot carriers driven by the temperature gradient and cold carriers driven by the Seebeck electric field tending to cancel each other out (Fig. 5C) (*30*). However, in an ambipolar, two-carrier system, unlike *R_H_* and *S*, for which electron and hole contributions will cancel each other out, υ should be enhanced as electrons and holes will move to opposite directions in a magnetic field, i.e., the contributions from electrons and holes to the transverse voltage add up (Fig. 5D). Consequently, the Nernst effect provides another independent way to confirm the existence of ambipolar transport. In CuBHT, the magnitude of υ gradually decreases with temperature but a clear peak is observed (at which the slope of ∆*V_xy_*/∆*T* versus magnetic field is highest). This peak occurs very close to the temperature (~100 K) where *R_H_* ~ 0 (Fig. 5, A and B). The observation of a peak in υ, at the point where the contributions of holes and electrons to *R_H_* cancel each other out, is a clear, characteristic feature of the ambipolar Nernst effect. Similar phenomena have been reported in several inorganic ambipolar systems including NbSe_2_ and CsV_3_Sb_5_ (*31*, *32*). The similarity in υ versus *T* between CuBHT and other known ambipolar inorganic materials is another strong confirmation that electrons and holes coexist in CuBHT. The measured Nernst coefficient in CuBHT is negative (Fig. 5B). The sign of the Nernst coefficient can be interpreted in terms of the relative contributions of hot carriers diffusing from the hot to the cold side and cold carriers drifting under the influence of the Seebeck electric field from the cold to the hot side. If the Nernst coefficient is dominated by the diffusion of hot carriers, a positive sign is expected (schematic diagram shown in Fig. 5D). On the other hand, if the Nernst effect is dominated by the drift of cold carriers due to the Seebeck electric field, a negative sign would be observed, which is the case for CuBHT. This indicates that the energy dependence of the mobility is negative in CuBHT (dμ/d*E* < 0).

### Two-carrier analysis

Summarizing our experimental characterizations, the sign reversals of *R_H_* and *S* and the peak in υ at the point at which *R_H_* ~ 0 all suggest that CuBHT is an ambipolar transport system with contributions of both electron and hole bands to electrical conduction. Therefore, a proper two-carrier analysis is needed to quantitatively extract electron and hole densities and mobilities from the measurements. We therefore constructed a simple two-band model that assumes a hole and an electron band with respective carrier concentrations *n_h_* (*n_e_*) and mobilities μ*_h_* (μ*_e_*) that are assumed to be field independent. In this model, the longitudinal resistivity (ρ*_xx_*) and the Hall resistivity (ρ*_xy_*) can be expressed as (*33*–*35*)

$$\rho_{\mathrm{xy}}=\frac{1}{e}\frac{\left(n_{h}\mu_{h}^{2}-n_{e}\mu_{e}^{2}\right)+\mu_{h}^{2}\mu_{e}^{2}B^{2}\left(n_{h}-n_{e}\right)}{{\left(n_{h}\mu_{h}+n_{e}\mu_{e}\right)}^{2}+\mu_{h}^{2}\mu_{e}^{2}B^{2}{\left(n_{h}-n_{e}\right)}^{2}}B$$ (3)

$$\rho_{\mathrm{xx}}=\frac{1}{e}\frac{\left(n_{e}\mu_{e}\mu_{h}^{2}+n_{h}\mu_{h}\mu_{e}^{2}\right)B^{2}+\left(n_{h}\mu_{h}+n_{e}\mu_{e}\right)}{{\left(n_{h}\mu_{h}+n_{e}\mu_{e}\right)}^{2}+\mu_{h}^{2}\mu_{e}^{2}B^{2}{\left(n_{h}-n_{e}\right)}^{2}}$$ (4)

Note that these equations are only valid in the classical regime (*T* > 30 K, no WL). This model has been successfully used to solve two-carrier problems in many inorganic materials, such as Dirac or Weyl semimetals (*34*, *35*), by simultaneously fitting ρ*_xx_* and ρ*_xy_* versus *B*. It is likely to be only a crude approximation of the real electronic band structure (to be discussed below), but a more realistic and complex model with multiple electron and hole bands crossing the Fermi level would contain too many free parameters that cannot all be determined by the measured experimental data.

Even for this simple model, the data are insufficient to determine the four unknown parameters. In principle, two parameters can be determined by fitting ρ*_xx_* versus *B* (one from the resistivity at *B* = 0 and the other from the curvature), whereas the remaining two parameters can be determined by fitting ρ*_xy_* versus *B* (one from the slope at *B* = 0 and the other from the curvature). In reality, however, we can only obtain one parameter from ρ*_xy_* versus *B* because, in CuBHT, the slope does not show any deviation from linearity within our ±14-T measurement range. The key difference between CuBHT and other inorganic materials is probably their mobilities considering that the nonlinear feature in ρ*_xy_* versus *B* usually occurs at *B* ~ μ^−1^. To observe any nonlinear feature at ~10 T, a mobility of ~1000 cm^2^ V^−1^ s^−1^ is needed. This condition is easily fulfilled in materials like Dirac or Weyl semimetals but not in CuBHT.

Therefore, an extra equation is needed to fully solve the two-carrier problem in CuBHT. For this, we use the Seebeck coefficient, which can also be expressed as a function of charge density. According to the Mott formula, in the free electron model, the electron Seebeck coefficient *S_e_* of a metal (which is the case for CuBHT) can be expressed in terms of the Fermi energy *E_F_* or Fermi temperature *T_F_* (*36*, *37*)

$$S_{e}=-\frac{\pi^{2}k_{B}}{3e}\frac{T}{T_{F}}$$ (5)

In a 3D free electron model, we can substitute $E_{F}=\frac{\hbar^{2}}{2m^{*}}{\left(3\pi^{2}n\right)}^{\frac{2}{3}}$ into Eq. 5, which results in

$$S_{e}=a\frac{T}{n^{\frac{2}{3}}}$$ (6)

Here, *a* is a constant that is equal to $-\frac{\pi^{2}k_{B}^{2}}{3e}\frac{1}{{\left(3\pi^{2}\right)}^{\frac{2}{3}}}\frac{2m^{*}}{\hbar^{2}}$, *k*_B_ is the Boltzmann constant, m^*^ is the effective mass, *n* is the carrier density, and ℏ is the reduced Planck’s constant. Equation 6 is valid for both electrons and holes, so we can get a similar expression for the hole Seebeck coefficient *S_h_*. We further assume that electron and holes have the same effective mass; this is an assumption for which we provide evidence below based on band structure calculations. The total Seebeck coefficient for a two-carrier system can then be written as

$$S=\frac{S_{h}\sigma_{h}+S_{e}\sigma_{e}}{\sigma_{h}+\sigma_{e}}$$ (7)

where σ*_e_* and σ*_h_* refer to electron and hole conductivity and $\sigma =\sigma_{e}+\sigma_{h}=e\left(n_{e}\mu_{e}+n_{h}\mu_{h}\right)$. Therefore, Eq. 7 can be rewritten as

$$S=\mathrm{aT}\frac{{\left(n_{e}\right)}^{\frac{1}{3}}\mu_{e}-{\left(n_{h}\right)}^{\frac{1}{3}}\mu_{h}}{n_{e}\mu_{e}+n_{h}\mu_{h}}$$ (8)

**Fig. 6.**
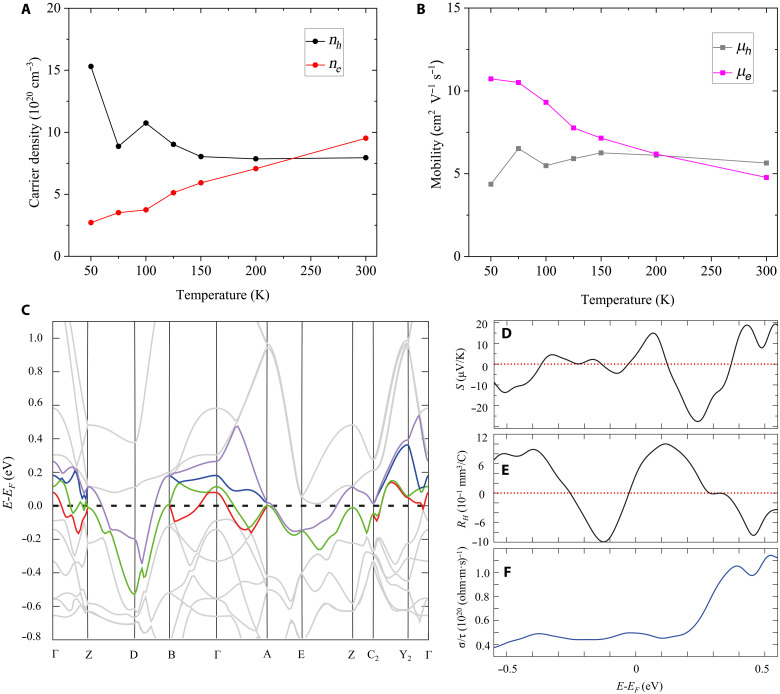
Two-carrier analysis. (**A**) *n_e/h_* and (**B**) μ*_e/h_* derived from free electron approximation at different temperatures. (**C**) Computed band structure at the DFT/PBEsol level of the CuBHT structure “**1**” (fig. S14, A and B). The four bands crossing the Fermi level (dashed line) are represented in red, green, blue, and purple color, whereas the others are set in gray scale for clarity. The color code is conserved for the visualization of the Fermi surface of the electron and hole pockets (see figs. S16 to S19). (**D**) Computed Seebeck coefficient in a ±0.5-eV window around the Fermi level at 300 K. A sign inversion is observed between −0.1 and +0.1 eV, i.e., in the energy window covered by the small electron and hole pockets described before. (**E**) Computed Hall effect in a ±0.5-eV window around the Fermi level at 300 K. A sign inversion is observed between −0.1 and +0.1 eV. (**F**) Computed electronic conductivity divided by the constant relaxation time in a ±0.5-eV window around the Fermi level at 300 K.

Now, we have enough equations to extract the four unknown parameters (*n_e/h_* and μ*_e/h_*) at each temperature. We start with assuming m^*^ being equal to the free electron mass. As shown in Fig. 6 (A and B), the results indicate that the contributions from electron and hole conduction are very close. For example, at 300 K, *n_h_* = 7.95 × 10^20^ cm^−3^, *n_e_* = 9.52 × 10^20^ cm^−3^, μ*_h_* = 5.65 cm^2^ V^−1^ s^−1^, and μ*_e_* = 4.78 cm^2^ V^−1^ s^−1^ are obtained. In addition, it is clear that *n_h_* and *n_e_* have different temperature dependences: *n_e_* is gradually depleted, whereas *n_h_* is slightly increasing with decreasing temperature. On the mobility side, μ*_h_* is nearly constant over a wide temperature range, whereas μ*_e_* experiences an increasing trend with decreasing temperature. The sign reversal of *R_H_* upon cooling can be explained by μ*_e_* becoming greater than μ*_h_* at low temperatures because mobility has a higher power in determining the sign of *R_H_* than charge density. It is also worth noting that the fitted values of μ*_e/h_* and *n_e/h_* are close to the mobility estimated from MR [*MR* ~ (μ*B*)^2^, assuming electrons and holes have the same mobility], as shown in fig. S12. The observed temperature dependence of the electron and hole parameters may arise from several factors. Intrinsically, because the DOS above and below the Fermi level are not symmetric, variations in temperature should induce a statistical shift of the Fermi level, modulating the electron and hole concentrations. In addition, the structure of CuBHT might undergo subtle temperature-dependent changes (e.g., changes in lattice parameters or strain), which could alter the band structure, thereby affecting carrier densities and mobilities. Extrinsic defects may also play an important role in influencing the temperature dependence of electron and hole parameters. At present, we are unable to distinguish between these factors, but the physical origin of the temperature dependence of the electron and hole concentrations and mobilities should be investigated in future work.

The extracted values of the four parameters depend of course on the assumed value of m*. To estimate how strong this dependence is, we also extracted these parameters assuming higher and lower values of m* (fig. S13). The temperature dependences of the four parameters with higher and lower m* values look similar, and there is no large difference in their mobility values (5 to 10 cm^2^ V^−1^ s^−1^) and carrier concentrations (5 × 10^20^ to 10 × 10^20^ cm^−3^). We also tried to use a 2D free electron model instead of 3D for the analysis (section S4.2) but failed to find solutions that fitted the experimental results. The failure of the 2D analysis may suggest that CuBHT is not a purely 2D electronic system; this is consistent with recent reports of the crystal structure of CuBHT not being a 2D, layered van der Waals structure but a more 3D structure with covalent bonds formed between the layers (*38*) (see discussion below).

Our two-carrier analysis results also provide a possible explanation for the inconsistent Seebeck results reported in the literature. In most papers, merely room temperature values of *S* are reported, and based on the sign of *S* at room temperature, the carrier type is claimed to be p-type or n-type. However, it is important to note that the observation of a positive (negative) sign of *S* does not exclude the existence of electrons (holes) but only means that holes (electrons) contribute more than electrons (holes). Our results suggest that, in CuBHT, there is a delicate balance where electrons and holes contribute almost equally; thus, any subtle change in material properties and/or Fermi level position would affect the experimentally obtained *S*. To note, as we mentioned above, the magnitude of *S* we measured here should be treated as an upper limit, and the true value of *S* should be smaller, which indicates even more balanced contributions from electrons and holes. The sign reversal upon cooling can be caused by different temperature dependences of *n_e/h_* and μ*_e/h_*. The quality of CuBHT film is sensitive to synthetic conditions, and even devices made by the same group can exhibit quite different charge transport behavior (*17*, *19*, *23*). Therefore, it is very likely that CuBHT films with similar conductivity values from different groups have slightly different levels of imperfection (e.g., defects and impurities), which may moderately shift the Fermi level and/or modify the electronic structure. Consequently, depending on the nature of the imperfection (which is difficult to predict), the relative contribution from either type of carriers can be enhanced/suppressed, explaining the inconsistencies observed in the literature.

We lastly interpret our results within the full, calculated band structure of CuBHT. These calculations were based on two reported crystal structures of CuBHT. One is the above mentioned, recently reported, non–van der Waals structure (referred to as structure “**1**”) obtained from CuBHT single crystals (*38*) (fig. S14), whereas the other is the older, widely reported monoclinic structure (referred to as structure “**2**” in the following) from ref. (*19*) (fig. S21). The quality of our structural characterization reported in Fig. 1 does not allow us to distinguish between the two structures [note that, if we had used the recent, non–van der Waals structure for the indexing of the GIWAXS pattern, the out-of-plane diffraction at *q* ~ 1.84 Å^−1^ would be indexed as (002)]. Theoretical calculations were performed (i) to validate, as far as this is possible, the assumptions made in our simplifying two-carrier model and (ii) to provide an independent prediction of the transport coefficients at room temperature based on the actual band structure. In the main text, we show the results for structure “**1**”, but structure “**2**” also gives similar results (section S5.2). The computed band structures (Fig. 6C) show multiple bands crossing the Fermi level at several high-symmetry points. This clearly supports our above observation/conclusion that electron and hole pockets must coexist in CuBHT at the Fermi level. Our above assumption of one electron and one hole band is clearly simplistic as there are four bands crossing the Fermi level and the corresponding Fermi surface has a complex topology. The analysis of the Fermi surfaces (see figs. S15 to S18) reveals multiple electron/hole pockets with a ball-like shape centered at the Γ point and extending over all k-paths in the CuBHT plane. Smaller droplet-like pockets are localized along the interlayer direction only. However, the relevant bands exhibit notable dispersion in both the intralayer and interlayer directions, providing some justification to our assumption of an isotropic effective mass on the order of the free electron mass. The calculated DOS also shows symmetric feature around *E_F_*, which further confirm near-equal conduction contribution from electrons and holes (fig. S19). A simple two-carrier model, which contains sufficiently few parameters to allow fitting to the available experimental transport data, will never be able to accurately reflect the full band structure. However, we feel that the calculated band structure provides sufficient justification for the assumptions made. Our simple two-carrier model should therefore provide useful order-of-magnitude information on the carrier concentrations and mobilities of electrons and holes in CuBHT.

We further used the calculated band structure to predict the transport coefficients within a Boltzmann transport formalism. The computed Seebeck coefficient at room temperature is very small at *E_F_* (<5 μV/K) and shows a sign inversion from (*E*_F_ − 0.1) eV to (*E*_F_ + 0.1) eV (Fig. 6D). This narrow energy window contains also the points in which the interlayer electron and hole pockets are localized. In the experimental film growth procedure, we have not included any electrolytes or salts; we therefore assumed in the calculations that our CuBHT unit cell is charge neutral and does not contain counterions. However, there may be small amounts of doping due to unintentional impurities, such as oxygen, and this is why it makes sense to consider not just the Fermi surface at *E*-*E_F_* = 0, but in a small energy window around *E*-*E_F_* = 0, when comparing with the experimental results. The computed Hall coefficient in this energy window also remains very small (Fig. 6E) (<1 mm^3^/C) with sign fluctuation at different energy levels. These small values of *S* and *R_H_* are in good agreement with our observations. The maximum Seebeck value in the electron pocket domain is on the order of 10 μV/K, which is comparable to that of hole pocket domain as shown in Fig. 6D. The computed conductivity, divided by the constant relaxation time (σ/τ), shows a symmetric profile around the Fermi level, supporting an assumption of similar mobilities for electrons and holes (Fig. 6F), which is also consistent with our finding from the two-band model. In a wider energy window (fig. S20), it is more obvious that both Seebeck and Hall coefficients fluctuate around 0, indicating comparable contribution from electrons and holes. There are points at which the predicted Seebeck coefficients are substantially larger. If it was possible to shift the Fermi level sufficiently by doping, this could potentially provide a means for enhancing the Seebeck coefficient and achieve higher levels of thermoelectric performance in CuBHT.

## DISCUSSION

We have investigated the charge transport physics of CuBHT films with a high electrical conductivity ~1450 S/cm at room temperature. The films exhibit metallic transport over a wide temperature range, whereas at low temperatures, WL and EEI effects lead to a drop of conductivity. Temperature-dependent Hall effect and Seebeck measurements clearly indicate ambipolar transport behavior of CuBHT as the signs of both *R_H_* and *S* change upon cooling. The ambipolar characteristics have been further confirmed by a peak in Nernst coefficient when contributions from electrons and holes are compensated. Using a simple, two-band model both carrier densities and mobilities of electrons and holes can be derived at different temperatures, revealing very similar contributions from electron and hole conduction (for example, the ratio between hole conductivity and electron conductivity is 0.99 at 300 K). Our work indicates that care must be taken when determining carrier polarity and extracting carrier parameters in conducting coordination polymers, and measurements of multiple transport coefficients are strongly suggested to better understand the charge transport properties in those materials and obtain reliable estimates for carrier concentrations and mobilities. Such detailed analysis of the transport physics is necessary to obtain reliable transport parameters, such as carrier mobilities and carrier concentrations, and to identify strategies for enhancing the thermoelectric transport coefficients of these CONASHs.

## MATERIALS AND METHODS

### Materials

Copper acetate (CuAc_2_) was purchased from Sigma-Aldrich with 99.99% trace metals basis. CF {assay > 99.9%, water < 0.0025%, and residue < 0.0001%, with amylene as a stabilizer [~25 parts per million (ppm)]} was purchased from Romil Co. Ltd.

### Synthesis of BHT

The synthesis of BHT basically follows ref. (*39*). Fourteen milliliters (112 mmol) of benzyl mercaptan was added to 3.4 g (85 mmol) of sodium hydroxide powder dispersed in 50 ml of *N*,*N*-dimethylformamide, followed by gradual addition of 3.4 g (12 mmol) of hexachlorobenzene (C_6_Cl_6_). The mixture turned orange, and the reaction proceeded exothermically. The mixture was kept stirred until it cooled down to room temperature. The mixture was dissolved in 180 ml of CF, followed by the addition of 400 ml of methanol to obtain C_6_(SBn)_6_ as a yellow precipitate. It was filtered, washed with methanol, and dried in vacuo (5.789 g, 60%). The following processes were done by the Schlenk technique. An excess amount of sodium (~2 g) was dissolved in liquid ammonia (~100 ml) cooled in dry ice/acetone, followed by the careful addition of C_6_(SBn)_6_ (2.177 g, 2.688 mmol). It was stirred for 4 hours and quenched by the careful addition of degassed methanol (20 ml). The solution was gradually warmed to room temperature. Degassed water (20 ml) was added and washed repeatedly with degassed diethyl ether in the Schlenk flask. Degassed hydrochloric acid (5 wt %, 100 ml) was added to obtain BHT as an off-white precipitate. The precipitate was filtered, washed with methanol, and dried in vacuo (642 mg, 88%). BHT was stored under an inert atmosphere. ^1^H NMR (500 MHz, CDCl_3_, ppm): 4.82 (s, 6H). Nuclear magnetic resonance (NMR) was measured with JNM-ECZ500 (JEOL Ltd.)

### Synthesis of CuBHT

#### Solution preparation

CF and distilled water were degassed by continuous N_2_ flow. CF was degassed for 1 hour, and water was degassed overnight. After degassing, both solvents were transferred to a glove box immediately. After that, 3 mg of BHT was dissolved in 10 ml of CF with a 20-ml vial. The solution was stirred at 45°C overnight in a N_2_-filled glove box. CuAc_2_ was dissolved in water with a concentration of 10 mM.

#### Synthesis

Six milliliters of CF was added into a 20-ml vial, and 1.2 ml of BHT/CF solution was added into a CF solvent by a glass syringe with a 0.45-μm polytetrafluoroethylene (PTFE) filter. Water (6.5 ml) was then added into the diluted BHT/CF solution gently by a plastic syringe with a 0.45-μm nylon filter. The vial was put in a hot plate with 45°C for 10 min. After that, 0.32 ml of CuAc_2_/water solution was added into the vial by a plastic syringe with a 0.45-μm nylon filter slowly and evenly around the bottle neck, keeping the syringe close to the bottle wall. We left the vial on the hot plate at 45°C for 2 hours. A dark blue film would be seen after several minutes.

After the reaction, the vial was opened, and 3 ml of water phase was removed by a glass pipette. Another 6.5 ml of water was added into the water phase by a plastic syringe with a 0.45-μm nylon filter slowly and evenly around the bottle neck, keeping the syringe close to the bottle wall, and then ~6 ml of water was removed. This washing process was repeated five times. After that, a substrate was held upside down (surface with electrodes was facing down) by a pair of tweezers and moved slowly through water and water-CF interface to the bottom of the vial. The water was removed completely by a glass pipette while holding the tweezers with a substrate steady and still. When the interface disappeared, we moved the tweezers slowly out of the solution and then put the substrate on a tissue gently. After about 10 min, the substrate with the film on top would be dry and then it was transferred into a CF solvent for washing for 10 min. The substrate was taken out and left on a tissue until it was completely dried.

### Multifunctional device fabrication

The devices were fabricated on 0.7-mm-thick Corning 1737F glass substrates via photolithography. First, glass substrates were cleaned by sonication in a sequence of water, acetone, and isopropanol (5 min each) and then treated by O_2_ plasma for 10 min. After spin coating and photolithography, substrates were transferred into a high-vacuum evaporator and Cr/Au (3/20 nm) electrodes were deposited by thermal evaporation. The substrates were then left in *N*-methyl-2-pyrrolidone overnight. After liftoff, all substrates were cleaned by isopropyl alcohol (IPA) and dried by N_2_ flow. The substrates were treated by O_2_ plasma for 10 min before CuBHT film transfer. After transferring the CuBHT film onto the device surface, the film on the undesired region of the device was first removed by tape and then transferred to a microscope working station for fine patterning.

### Structural and spectroscopic characterizations

GIWAXS was performed in Beamline I07 of Diamond Light Source. SEM and EDS were taken by an FEI Helios SEM/FIB. θ-2θ XRD was measured by a Bruker D8 Advance powder x-ray diffractometer. XPS and UPS were measured by a Thermo Fisher Scientific Escalab 250xi x-ray photoemission spectrometer. A Veeco Dimension 3100 atomic force microscope was used, operating in tapping mode, to assess the surface morphology and thickness of the polymer films.

### Magnetotransport measurements

Resistivity, Hall effect, and MR were measured using a Quantum Design physical property measurement system (PPMS) DynaCool system with a dc resistivity puck. The device was stuck on the puck with double-side copper tape and bonded with Al wires. All the measurements were taken using a 50-μA excitation current with a magnetic field step of 0.35 T from −14 to 14 T. More details are described in section S2.

### Thermoelectric measurements

The Seebeck coefficient was also measured in the PPMS chamber but using two external Keithley instruments. The temperature was fully controlled by PPMS. A Keithley 2612B source measure unit was used to supply the heater power, and a Keithley 2182 nanovoltmeter was used to measure the thermal voltage. The temperature gradient was determined from temperature resistance coefficients of thermometers at the hot and cold sides of film. For Nernst measurements, the transverse thermal voltage was measured at magnetic fields of −14, 0, and 14 T at the same heater power range. More details are described in section S3.

### Theoretical calculations

The primitive unit cells of the CuBHT structures (called structure “**2**” and “**1**” beforehand in the text) were extracted from the GIWAXS data, and their geometry were fully reoptimized at the density functional theory (DFT) level using the Vienna Ab initio Simulation Package (VASP) code (*40*, *41*). The projected augmented wave potential method was used with an energy cutoff of 520 eV to expand the plane waves. Electronic exchange and correlation were considered using the revised Perdew-Burke-Ernzerhof functional for solids (PBEsol) (*42*). The integration over the Brillouin zone was done using a (4 × 4 × 10) and (2 × 2 × 4) *k*-point Monkhorst-Pack grid for the relaxation of lattice vectors and atomic positions of the “**2**” and “**1**” structures, respectively. The primitive unit cell of structure “**2**” has a *C*2/*m* symmetry with final lattice parameters of *a* = *b* = 8.572 Å, *c* = 3.476 Å, α = β = 99.92°, and γ = 59.99°, in agreement with previously reported lattice models (*17*, *19*, *23*), whereas the primitive unit cell of structure “**1**” has a *P*2_1_/*c* symmetry with final lattice parameters of *a* = 14.744 Å, *b* = 8.589 Å, *c* = 6.401 Å, α = γ = 90.00°, and β = 91.32° (*38*). The relaxed geometry of these two structures were used to compute the electronic structure with an increased k-grid of (14 × 14 × 29) for structure “**2**” and (7 × 12 × 16) for structure “**1**” (*38*). The band structure was computed along the suggested k-path from the SeeK-path tool (*43*). The high-symmetry points localized in the CuBHT plane (structure “**2**”) are the *Y*, *C*, and *V* points, whereas Γ-*A*, Γ-*L*_2_, and Γ-*M*_2_ paths are along the interlayer direction (fig. S22C). The high-symmetry points for structure “**1**” are given in fig. S14C. Directions along the layer plane are oriented along the Γ-*Y*_2_ and Γ-*Z* paths, whereas the interlayer direction is along the Γ-*B* path. We used the XCrysden (*44*) and FermiSurfer (*45*) software to visualize the Fermi surfaces.

The transport properties (conductivity and Seebeck and Hall coefficients) were computed from the interpolation of the electronic structure coming from the native output of VASP using the BoltzTraP2 package (*46*). The transport coefficients are calculated based on the rigid-band approximation, which allows us to directly compute the carrier concentration from the interpolated DOS.

## Supplementary Materials

### This PDF file includes:

Sections S1 to S5 Figs. S1 to S28 Tables S1 to S3 References
