# Supplementary material for: Revealing the charge transport physics in metallic coordination nanosheets by thermoelectric and magnetotransport measurements
Source: Sci Adv. 2025 Apr 9;11(15):eadt9196. doi: 10.1126/sciadv.adt9196 (PMC11980846; doi:10.1126/sciadv.adt9196)
Supplement: Supplementary file 1 — Sections S1 to S5 Figs. S1 to S28 Tables S1 to S3 References [file sciadv.adt9196_sm.pdf]

Science Advances
AAAS

Supplementary Materials for

Revealing the charge transport physics in metallic coordination nanosheets by thermoelectric and magnetotransport measurements

Tian Wu *et al.*

Corresponding author: Xinglong Ren, xr216@cam.ac.uk; Henning Sirringhaus, hs220@cam.ac.uk

Sci. Adv. **11**, eadt9196 (2025)
DOI: 10.1126/sciadv.adt9196

This PDF file includes:

Sections S1 to S5
Figs. S1 to S28
Tables S1 to S3
References

Supplementary Materials

Section S1. Morphology and Structure

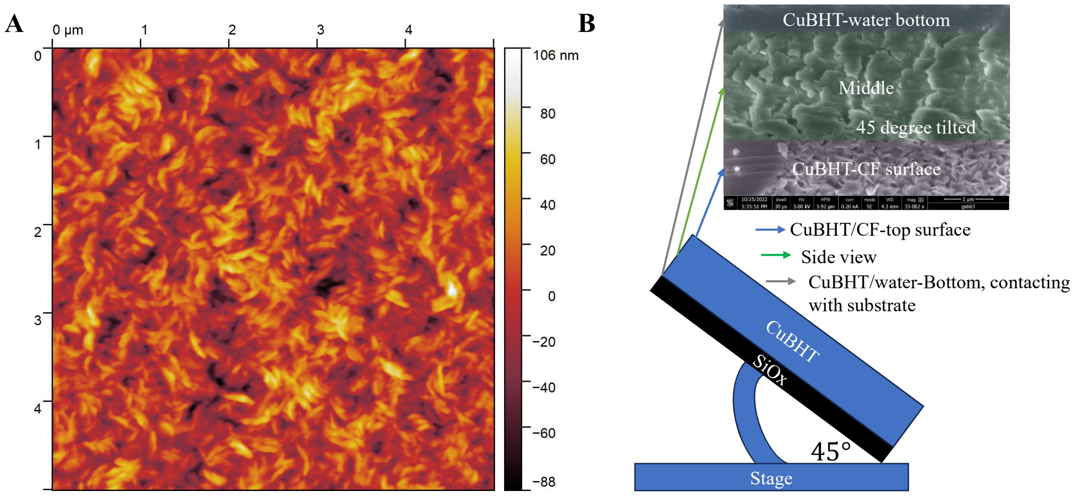

Fig. S1. Morphology of CuBHT films. (**A**) A 5 µm*5 µm AFM image of the top surface (rougher side) of a CuBHT film, deposited on a glass substrate; (**B**) Cross-section image of a CuBHT film obtained from a 45-degree titled stage.

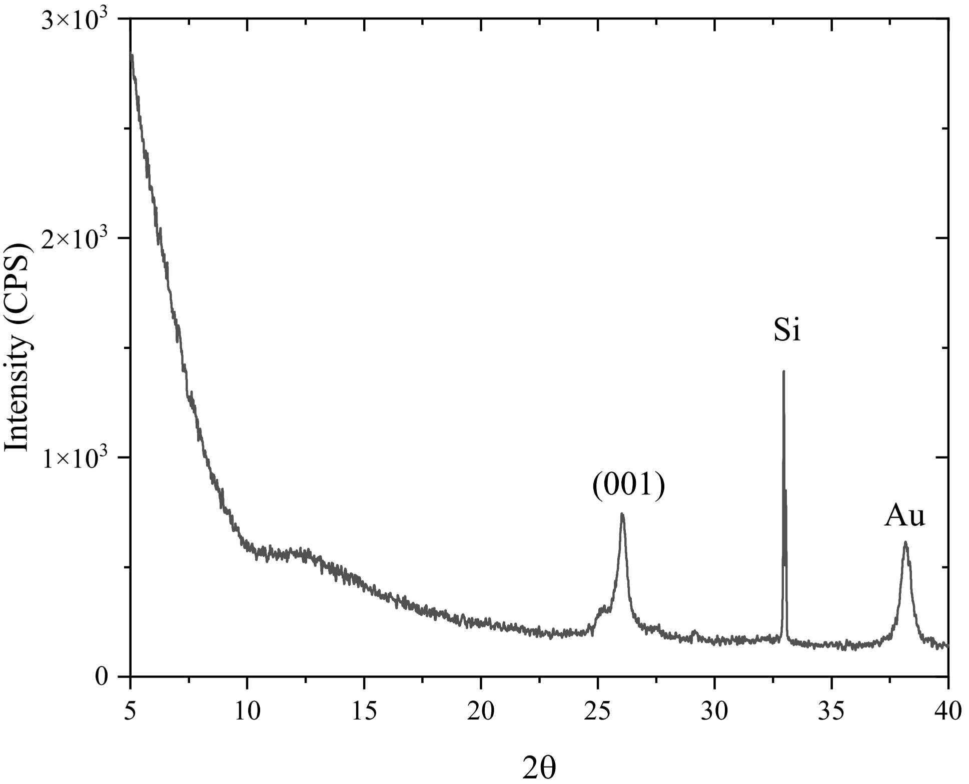

Fig. S2. Structural characterization of CuBHT. *θ-2θ* XRD of a CuBHT film deposited on a 1cm*1cm Si wafer.

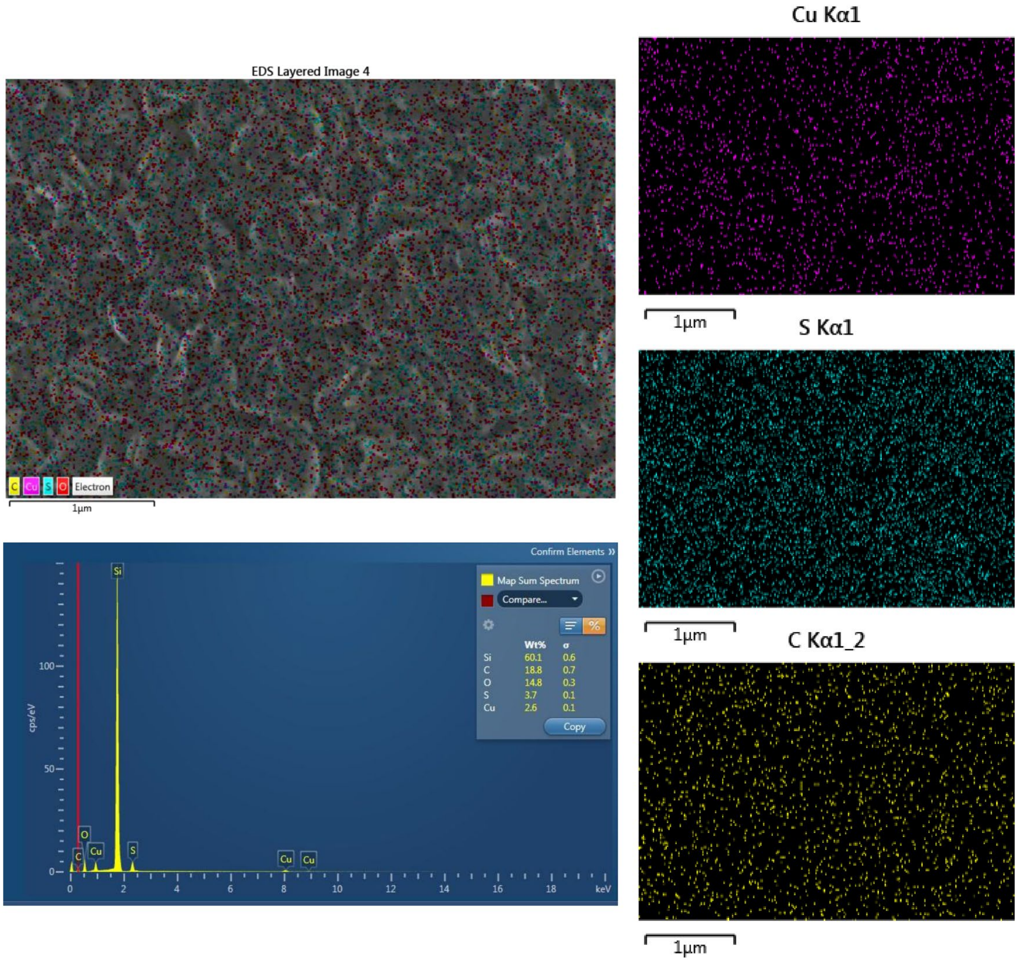

Fig. S3. Element analysis of CuBHT. EDS mappings of CuBHT with signals from Cu, S, C elements respectively. The EDS spectrum is shown in the bottom left.

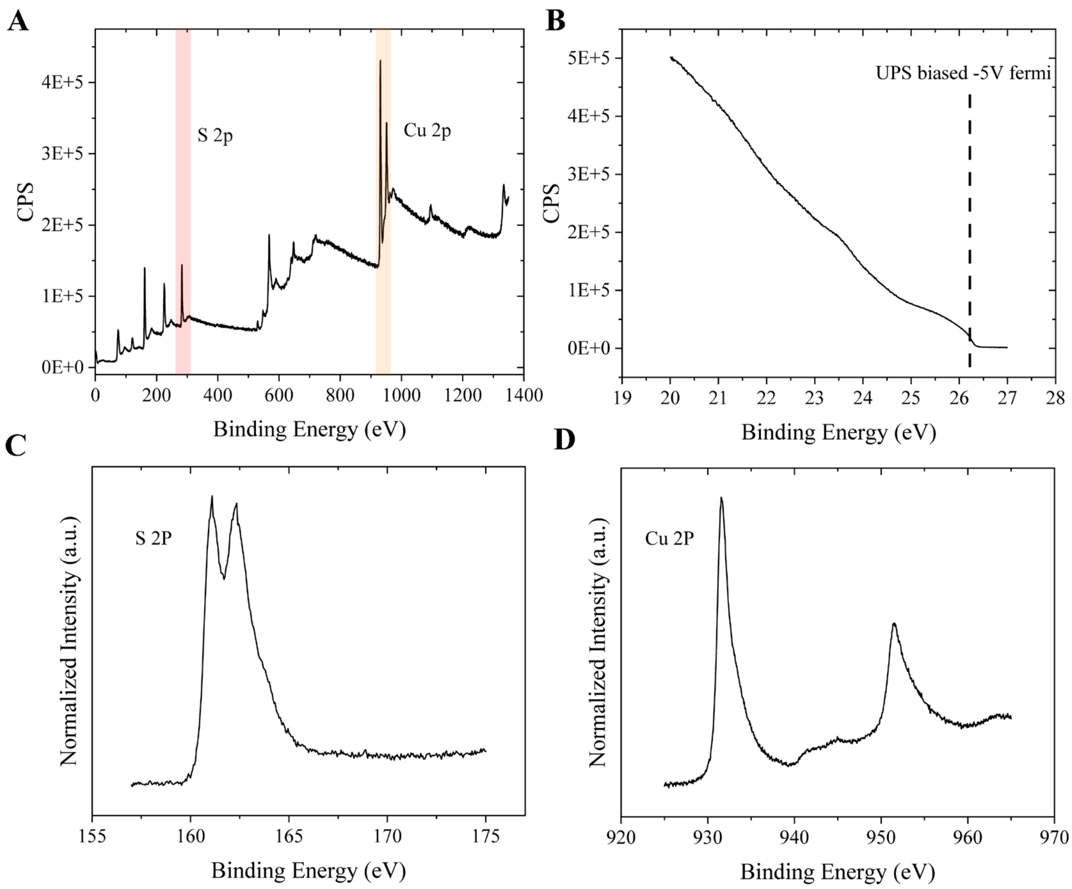

Fig. S4. X-ray and ultraviolet photoemission spectra of CuBHT. (**A**) Full XPS spectra of a CuBHT film on Si; (**B**) UPS spectra of CuBHT with -5V bias applied during the measurement; XPS spectra of (**C**) S 2p peaks and (**D**) Cu 2p peaks.

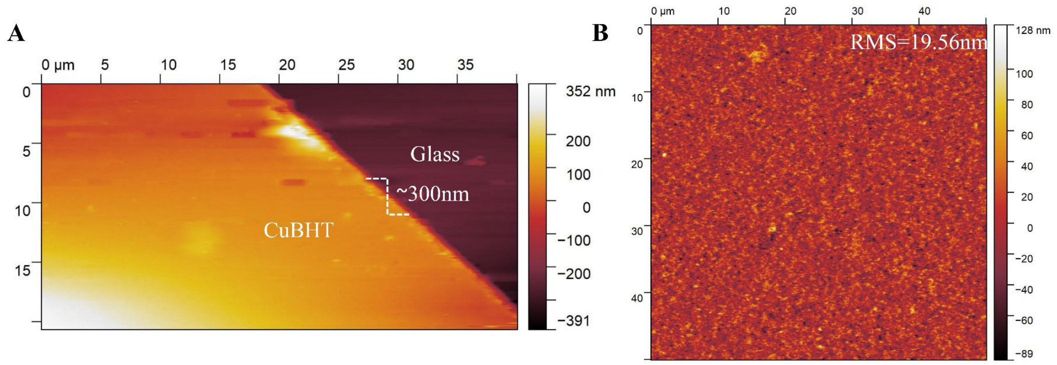

Fig. S5. Surface characterizations of CuBHT. (**A**). An AFM image with line profile from CuBHT to glass. (**B**). A 50μm*50μm AFM image of the top surface (rough side) of a CuBHT film on a prepatterned device.

Section S2. Electrical measurements

Resistivity, Hall effect and Magnetoresistance (MR) were measured using a Quantum Design Physical Property Measurement System (PPMS) DynaCool system with a DC resistivity puck. The device was stuck on the puck with double-side copper tape and bonded with Al wires. All the measurements were taken using a 50 μA constant current with a magnetic field step of 0.35 T from -14 T to 14 T.

Angular-dependent measurements were used to distinguish mechanisms behind observed MR results. The parabolic positive MR at 50 K disappears when the direction of magnetic field becomes parallel to the direction of current, consistent with the ordinary MR effect due to the Lorentz force. The negative MR at 10 K also becomes much smaller when the direction of magnetic field becomes parallel to the direction of current, indicating the 2D nature of weak localization (WL). In contrast, the positive component at 2 K does not change much with the direction of magnetic field and is likely due to electron-electron interaction (EEI). Additionally, the difference between MR at 90° and 0° at 2 K, or the anisotropic component, is negative and its shape resembles that of WL. Therefore, it is reasonable to conclude that 2D WL and EEI coexist at 2 K.

We also fitted the MR results at 15 K and 10 K (it is difficult to distinguish the contribution to MR from WL at other temperatures) with a 2D Hikami-Larkin-Nagaoka model (*47*), and the extracted phase coherence length (L_{φ}) is on the order of 10 nm (Table S1).

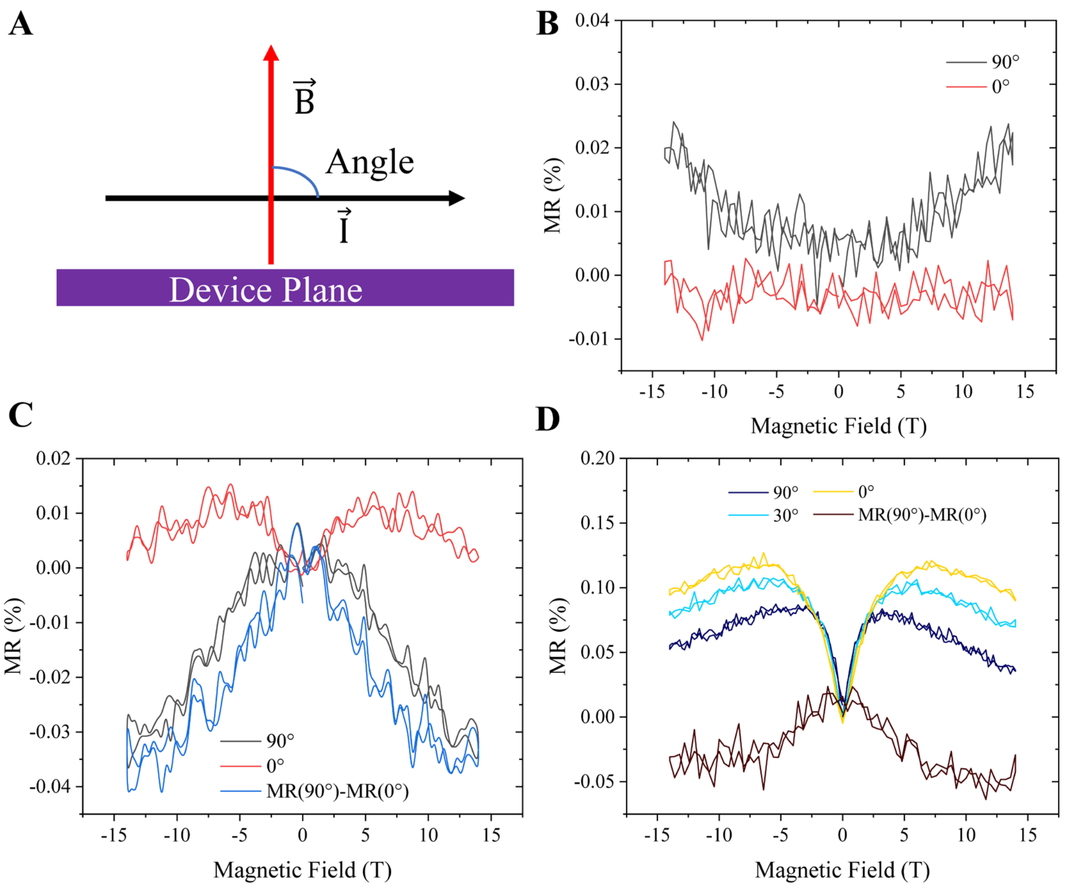

Fig. S6. Magnetoresistance measurements of a CuBHT device. (**A**) A schematic of angular dependent MR measurements. When the angle equals 90°, the magnetic field is perpendicular to the current flow through the CuBHT film while at 0°, magnetic field is parallel to the current; (**B**) MR measurements of CuBHT at 90° and 0° at 50 K; (**C**) MR measurement of CuBHT at 90° and 0° at 10 K and the difference between 90° and 0°; (**D**) MR measurements of CuBHT at 90°, 30°, and 0° at 2 K and the difference between 90° and 0°.

Table S1 Phase coherence length derived from 2D WL

Temperature (K)	L_φ (nm)
15	11.0
10	19.5
5	20.2
2	32.0

Section S3. Thermoelectric measurements

The Seebeck coefficient was also measured in the PPMS chamber but using two external Keithley instruments. A Keithley 2612B source-measure unit was used to supply the heater power and a Keithley 2182 nanovoltmeter was used to measure the thermal voltage. First, a current is supplied to the heater of the device to generate the temperature difference. After 15 s of stabilization, the nanovoltmeter was used to detect the voltage difference. 11 steps of heater power were used during the measurements and the thermal voltage scales linearly with heater power as shown in **Fig. S7**. The process is fully controlled by a homebuilt JAVA-based program (*48*). The slope of thermal voltage *vs* heater power is $\frac{\Delta V_{xx}}{\Delta P}$. Here ΔV_{xx} refers to the longitudinal voltage induced by the temperature gradient. To obtain the temperature difference between the hot side (close to heater) and cold side (far from heater) thermometers, the resistances of the electrodes were measured at the same heater power range with same steps. The results are shown in **Fig. S8**. The slopes of thermometer resistances vs heater power are $\frac{\Delta R_{hot}}{\Delta P}$ and $\frac{\Delta R_{cold}}{\Delta P}$. The temperature coefficients of hot and cold side thermometers are $\frac{\Delta R_{hot}}{\Delta T}$ and $\frac{\Delta R_{cold}}{\Delta T}$ respectively, which is calculated from the temperature dependent thermometer resistance measurements as shown in **Fig. S9**. The Seebeck coefficient can be calculated by

$$S = \frac{\frac{\Delta V_{xx}}{\Delta P}}{\frac{\frac{\Delta R_{hot}}{\Delta P}}{\frac{\Delta R_{hot}}{\Delta T}} - \frac{\frac{\Delta R_{cold}}{\Delta P}}{\frac{\Delta R_{cold}}{\Delta T}}} = \frac{\Delta V_{xx}}{\Delta T} \quad \text{(S1)}$$

As shown in **Fig. 4** in the main text, the magnitude of the measured Seebeck coefficients is small (about or below 1 μV/K). To figure out how much the measured Seebeck coefficients might be affected by experimental factors (e.g., thermal voltage contribution from Au electrodes), we performed a control experiment where the CuBHT channel was replaced by a 20 nm thick Au layer (**Fig. S10**). The difference between **Fig. 4** and **Fig. S10** should be the difference between the Seebeck coefficients of CuBHT and of Au. In terms of the magnitude, the measured values for the Au device (S_1) are generally smaller than those for the CuBHT device (S_2), and at some temperatures S_1/S_2 can be ~50%. Additionally, the reported Seebeck coefficient values for Au thin films are also on the order of 1 μV/K (*29*). These results suggest that the

magnitude of the measured Seebeck coefficient for CuBHT might include the contributions from other experimental factors and should be treated as the upper bound. For Nernst measurements, the transverse thermal voltage was measured at magnetic fields of -14T, 0T, 14T respectively at the same heater power range. A typical Nernst signal at different magnetic fields is shown in **Fig. S11** where ΔV_{xy} is the transverse voltage induced by the temperature gradient at a specific magnetic field. Similar with Seebeck coefficient calculation, the Nernst coefficient (ν) can be derived as:

$$\nu = \left(\frac{\frac{\Delta V_{xy}}{\Delta P}}{\frac{\frac{\Delta R_{hot}}{\Delta P}}{\frac{\Delta R_{hot}}{\Delta T}} - \frac{\frac{\Delta R_{cold}}{\Delta P}}{\frac{\Delta R_{cold}}{\Delta T}}}\right) \times \frac{1}{\Delta B} \times \frac{L}{W} = \frac{\Delta V_{xy}}{\Delta T \times \Delta B} \times \frac{L}{W} \quad \text{(S2)}$$

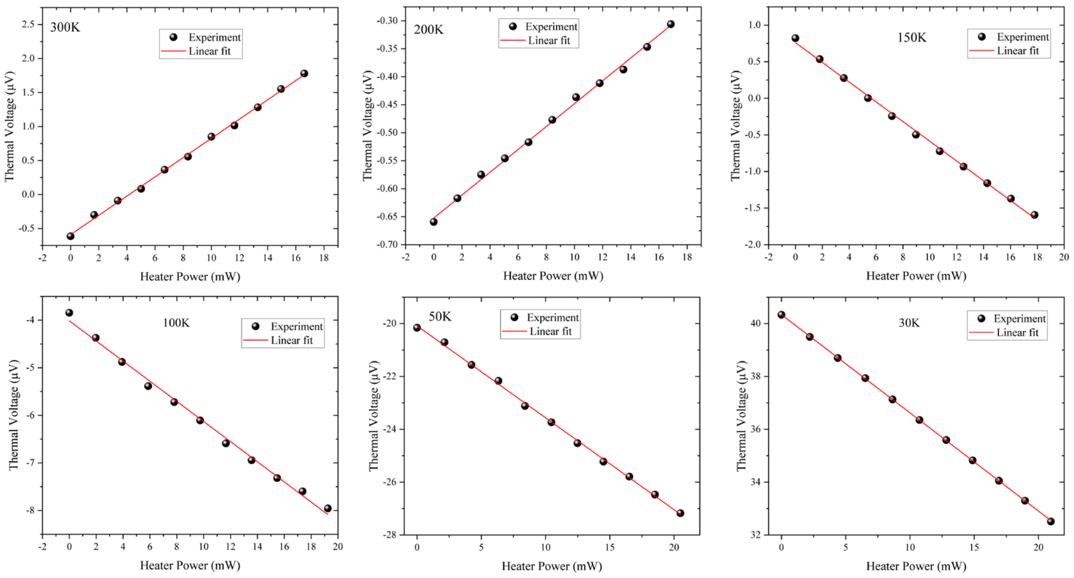

Fig. S7. Thermal voltage measurements. Experimental V_{xx} measured from different heater powers and linear fit of experimental data at 300 K, 200 K, 150 K, 100 K, 50 K and 30 K.

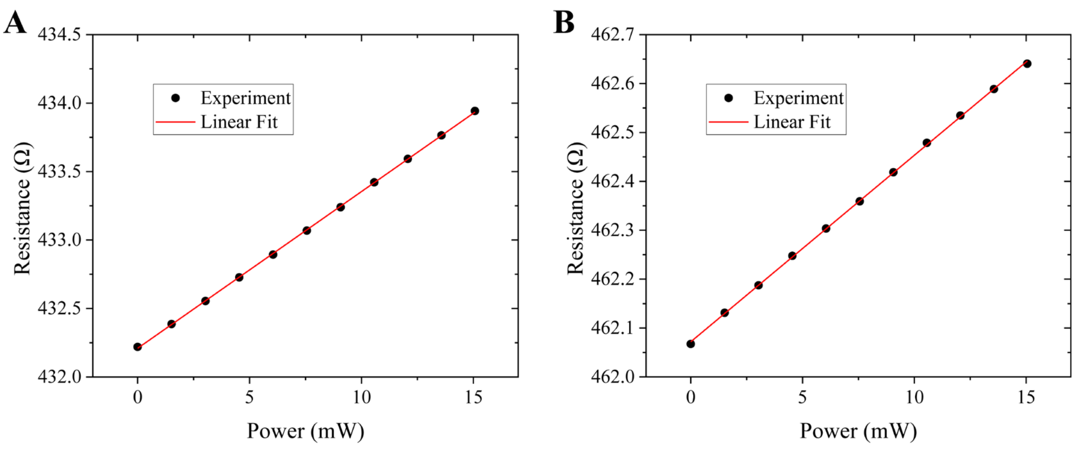

Fig. S8. Resistance changes of built-in thermometer *vs* heater power. (**A**) Change of hot side thermometer resistance with varying heater power; (**B**) change of cold side thermometer resistance with varying heater power. Red lines are a linear fit of experimental data. The temperature is 300 K.

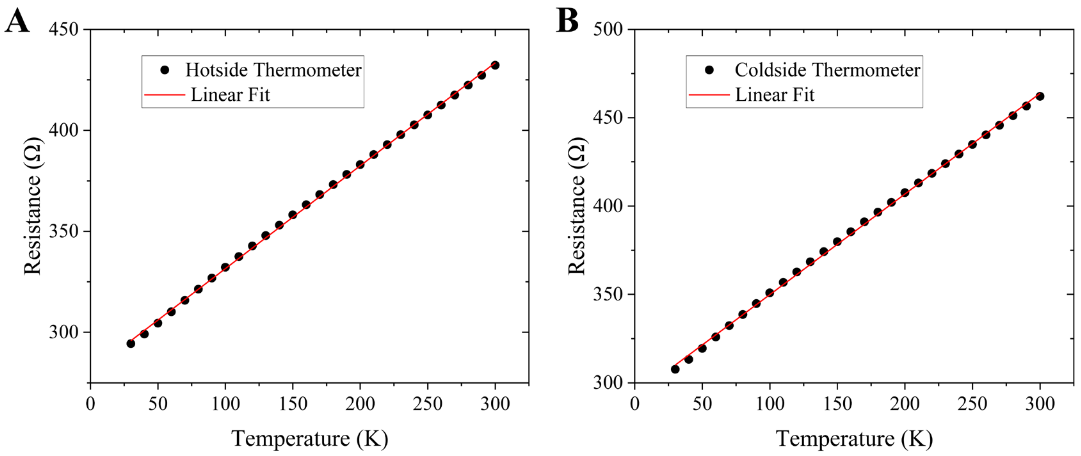

Fig. S9. Temperature dependent thermometer resistance. (**A**) Hot side thermometer and (**B**) cold side thermometer. Red lines are a linear fit of experimental data.

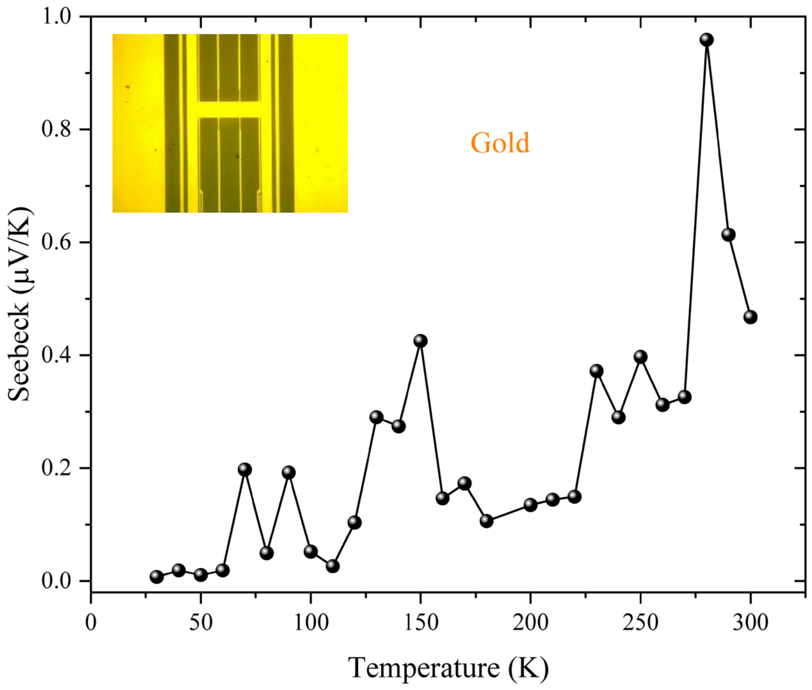

Fig. S10. Seebeck coefficient of gold. Temperature dependent Seebeck coefficient of a gold device on the same device architecture as CuBHT. Gold is thermally evaporated onto the same pre-patterned substrate as shown in Figure 2a with a thickness of ~20 nm (shown in the inset). In general, the measured Seebeck signal is smaller but comparable to that of CuBHT.

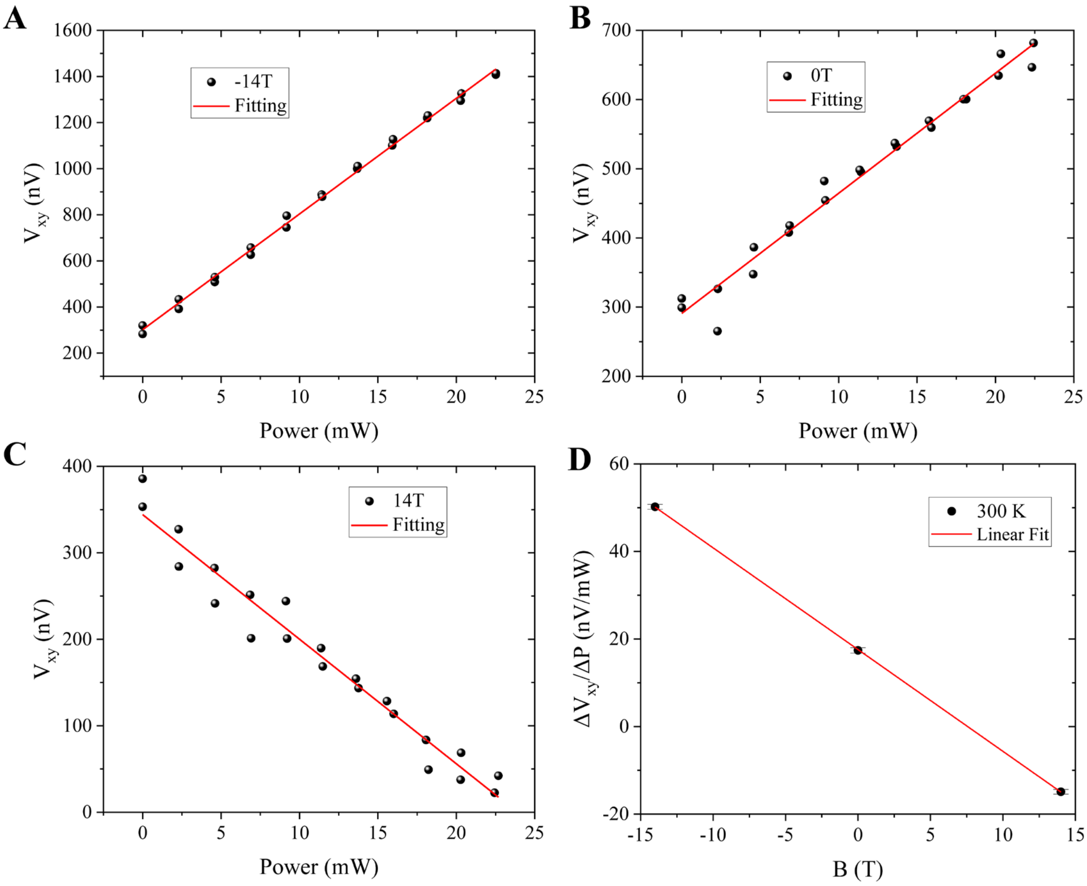

Fig. S11. V_{xy} measured at 300K at different the magnetic field. (**A**) -14T, (**B**) 0T and (**C**) 14T. Red lines are linear fits of experimental data; (**D**) Slope of thermal voltage *vs* heater power ($\frac{\Delta V_{xy}}{\Delta P}$) *vs* *B* at 300 K. The signal at 0T is non-zero due to the normal Seebeck effect caused by slight electrode misalignment.

Section S4. Extraction of electron/hole mobilities and carrier densities using two-band model

S4.1 Estimation of parameters from MR

The equations in the main text are solved numerically by MATLAB which requires an initial guess of the four parameters. The initial guess of four parameters is based on MR mobility which is derived from: $MR = (\mu B)^2$ assuming electron and hole have same mobility. The calculated mobilities at different temperatures are shown in **Fig. S12A** and the derived carrier densities are shown in **Fig. S12B**. Based on the 3D free electron model, the Seebeck coefficients calculated using MR-derived parameters are shown in **Fig. S12C**. The Seebeck values are in a reasonable range compared with experimental ones which indicates our initial guess based on MR mobility approximation is reasonable.

S4.2 Seebeck coefficient in the 2D free electron model

In a 2D system where the DOS is energy-independent, E_F can be written as:

$$E_F = \frac{\hbar^2 \pi n}{m^*} \tag{S3}$$

Then, S can be expressed as:

$$S_e = a\frac{T}{n} \tag{S4}$$

Here, a is also a constant which is equal to $-\frac{\pi^2 k_B^2}{3e}\frac{m^*}{\hbar^2}$, k_B is the Boltzmann constant, m^* is the effective mass, n is the carrier density, and $\hbar$ is the reduced Plank constant. For an ambipolar system, substituting **Equation S4** of both electron and hole to **Equation 7**:

$$S = aT\frac{\mu_e - \mu_h}{n_e\mu_e + n_h\mu_h} \tag{S5}$$

Applying the 2D model to our analysis, however, did not lead to any solution. We tried to fit the MR and Hall data using **Equations 3 and 4** and obtained multiple sets of possible fitting parameters ($\mu_{e/h}$ and $n_{e/h}$), but none of these sets of values gives the experimentally measured Seebeck coefficients. As shown in **Table S3**, even the maximum values calculated from the 2D model are more than 1 order of magnitude smaller than the experimental values. These results indicate that the 2D free electron model is not a good approximation for CuBHT, probably due to the existence of band dispersion along the π-π stacking direction.

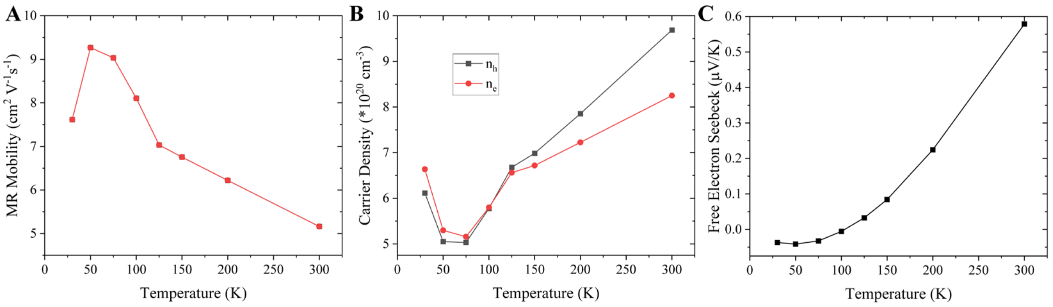

Fig. S12. Initial guess of transport parameters. (**A**) Temperature dependent mobility estimated from MR; (**B**) Derived $n_{h/e}$ values at different temperatures; (**C**) Calculated temperature dependent Seebeck coefficient under free electron approximation.

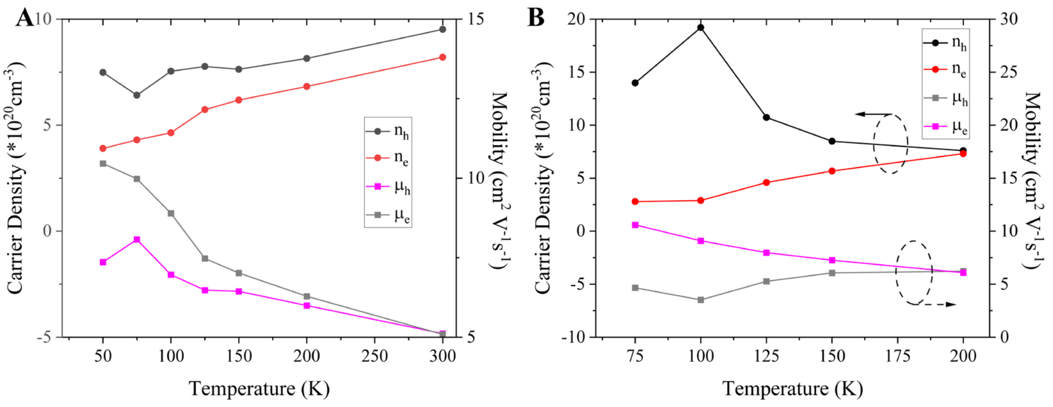

Fig. S13. Extracted charge density and mobility values at different effective mass. (**A**) Temperature dependent $n_{e/h}$ and $\mu_{e/h}$ solved by giving an effective mass of $2m_e$; (**B**) Temperature dependent $n_{e/h}$ and $\mu_{e/h}$ solved by giving an effective mass of $2/3m_e$. It is found that a m^* lower than m_e leads to no solutions for some temperatures while a m^* higher than m_e can still give reasonable values over the experimental temperature range.

Table S2 Calculated Seebeck coefficients at different temperatures (maximum absolute value) using a 2D free electron model.

Temperature (K)	*Max. S* (nV/K)
300	51.48
200	35.37
150	-33.58
100	-27.31
50	-14.95
30	-11.99

Section S5. Theoretical description of the CuBHT electronic structure

S5.1 Results for structure “1”

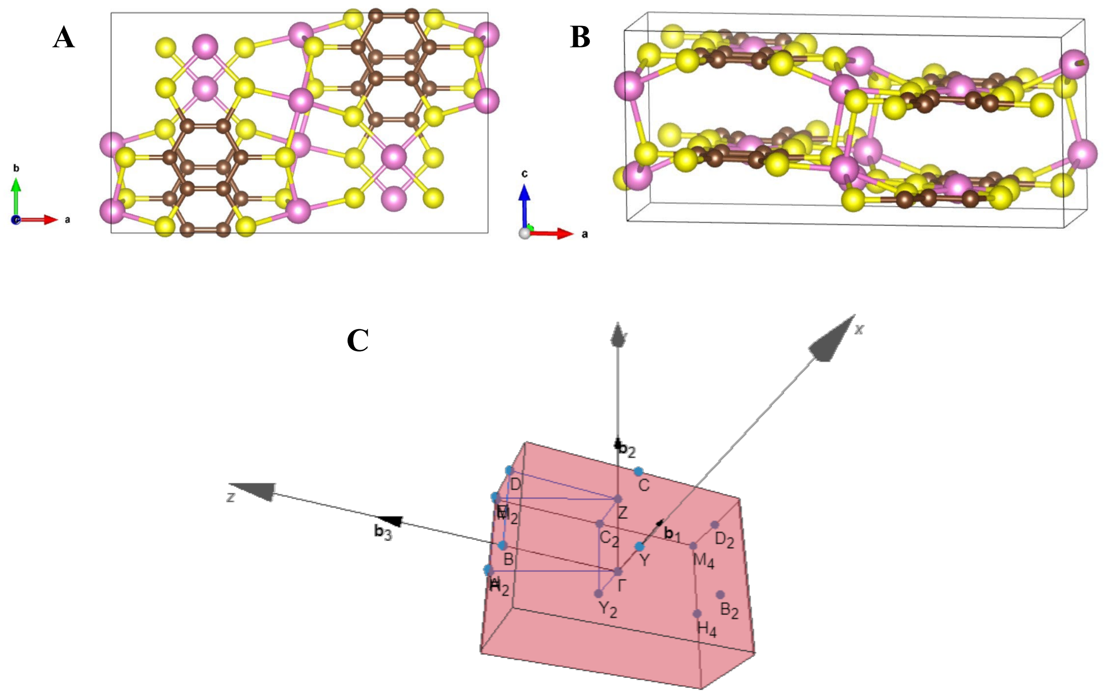

Fig. S14. Relaxed structure of the CuBHT primitive unit cell. The structure is based on a recently reported CuBHT structure (*38*) from top (**A**) and side (**B**) view. Carbon, sulfur and copper atoms are represented in brown, yellow and pink, respectively; (**C**) Scheme of the primitive brillouin zone with the position of the high symmetry point explored for the band structure calculation. Results in Figures **S15-S20** are based on this structure.

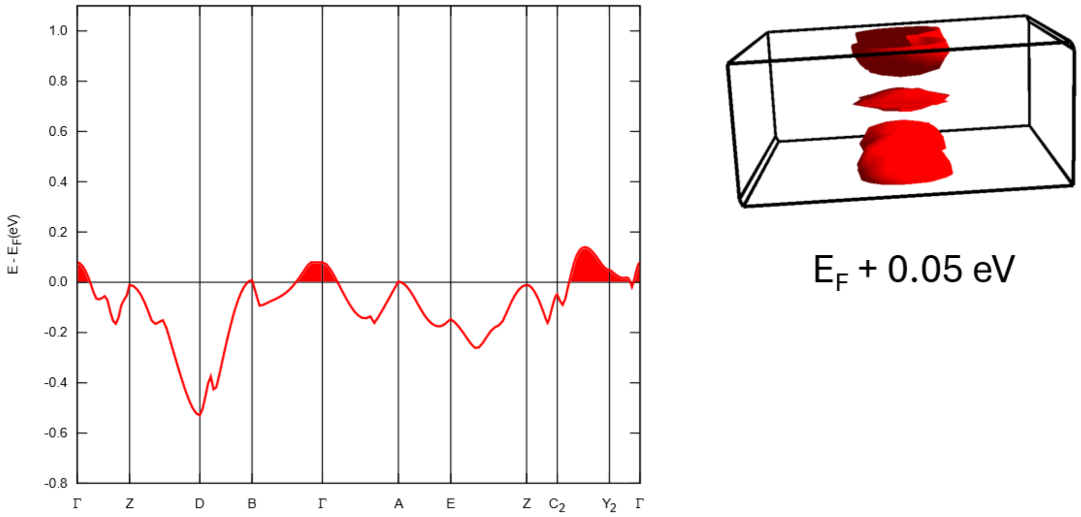

Fig. S15. Band structure of the first crossing band with fermi surface at 0.05 eV above the fermi level. The hole pockets are mainly centered at the Γ point and along the C_2-Y_2-Γ-Z path i.e. the in-plane direction.

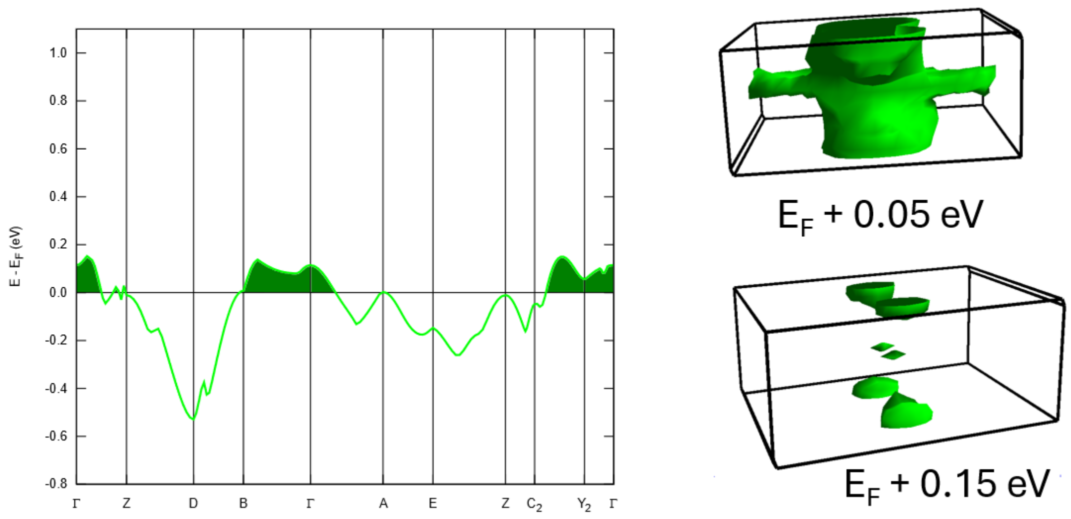

Fig. S16. Band structure of the second crossing band with fermi surface at 0.05 and 0.15 eV above the fermi level. The hole pockets are similar to the first crossing band (red, **Figure S15**) with, however, a larger expansion along the Γ-B path i.e. the direction along CuBHT interlayer. The occupied region of this band merges with the first crossing one along the Z-D-B and A-E-Z-C_2 paths.

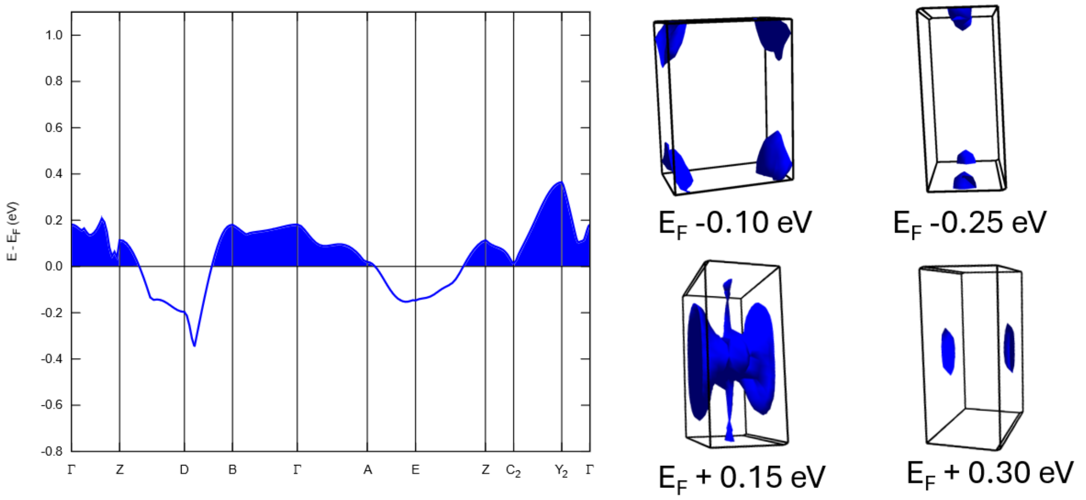

Fig. S17. Band structure of the third crossing band. The hole pocket shows a dispersion all over the brillouin zone. The electron pocket is mainly centered at the D and E point i.e. at the center of an edge.

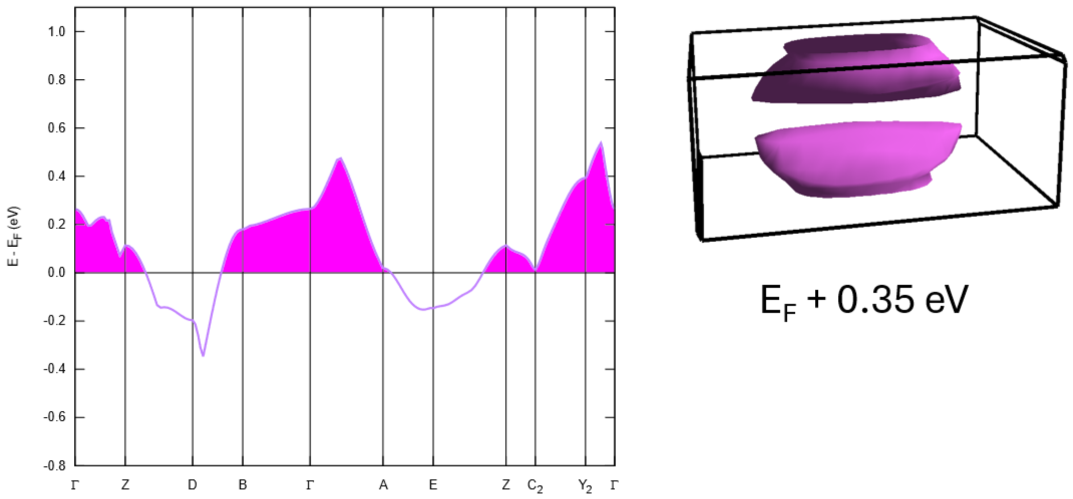

Fig. S18. Band structure of the fourth crossing band. This band merges with the previous one (**Fig. S17**, blue) all over the brillouin zone (occupied and unoccupied zone).

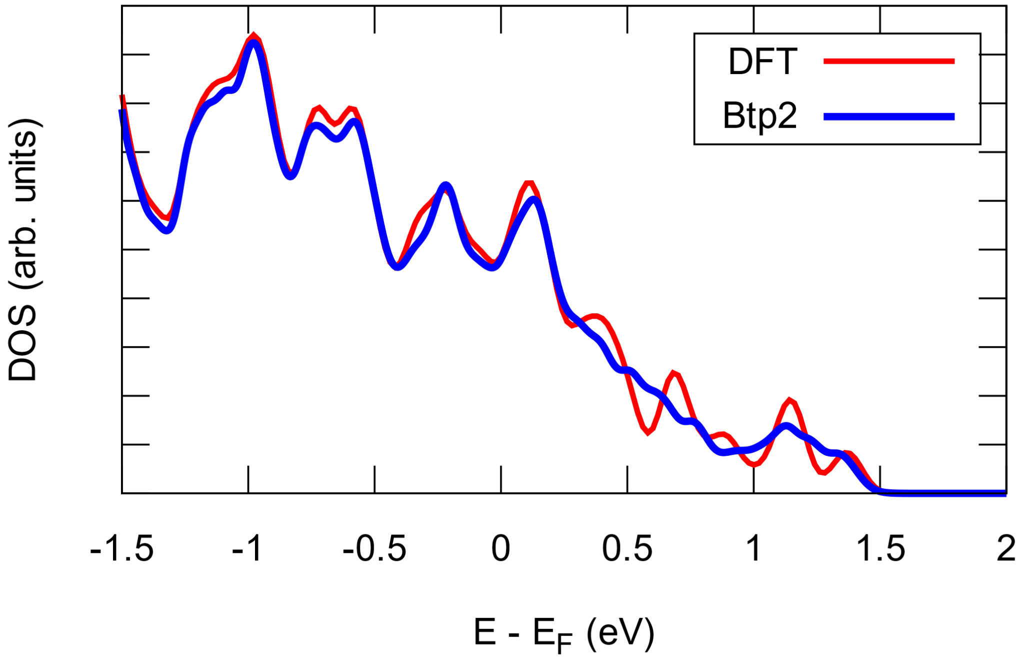

Fig. S19. Energy dependent density of states. Comparison between the interpolated density of states (DOS) obtained with the BolzTrap2 code (blue line) with the DFT result (red line) around the fermi level based on structure "**1**". The fluctuation of the DOS is nicely reproduced all along the investigated energy window.

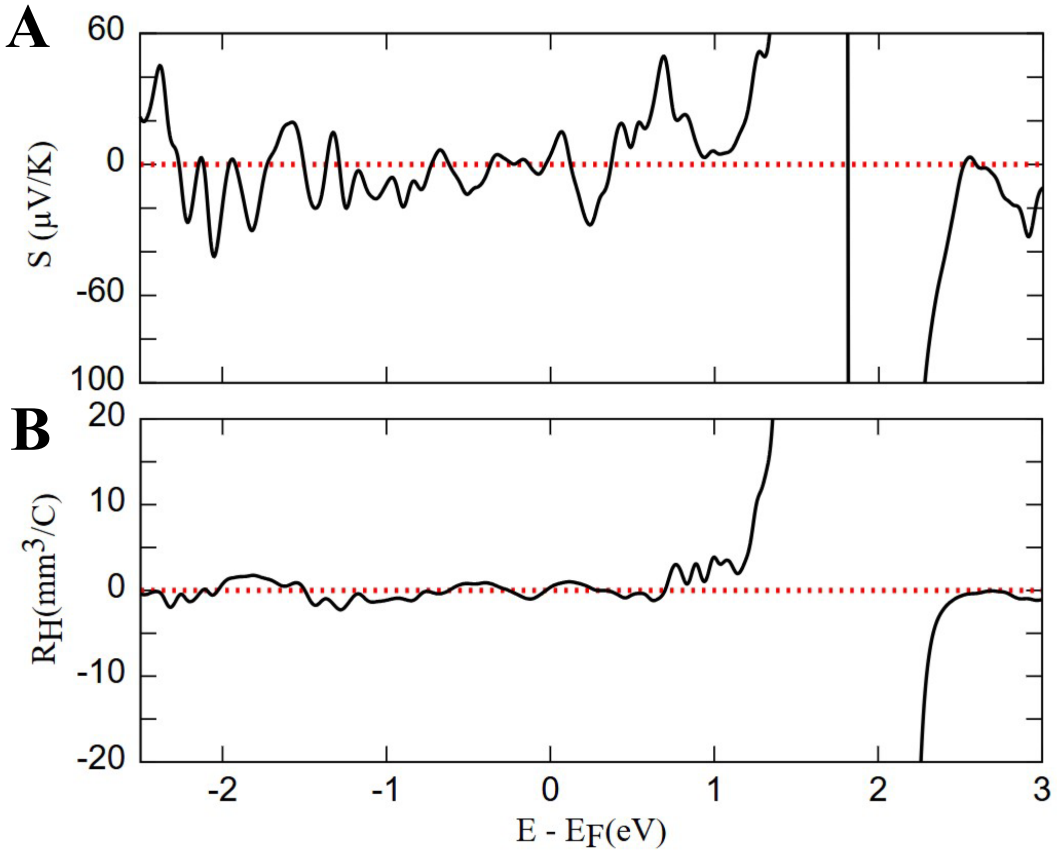

Fig. S20. Computed transport coefficients. (**A**) Computed Seebeck coefficient in a wider energy window (-2.5-3.0 eV) around the Fermi level at 300K; (**B**) Computed Hall effect in a wider window (-2.5-3.0 eV) around the Fermi level at 300K.

S5.2 Results for structure "2"

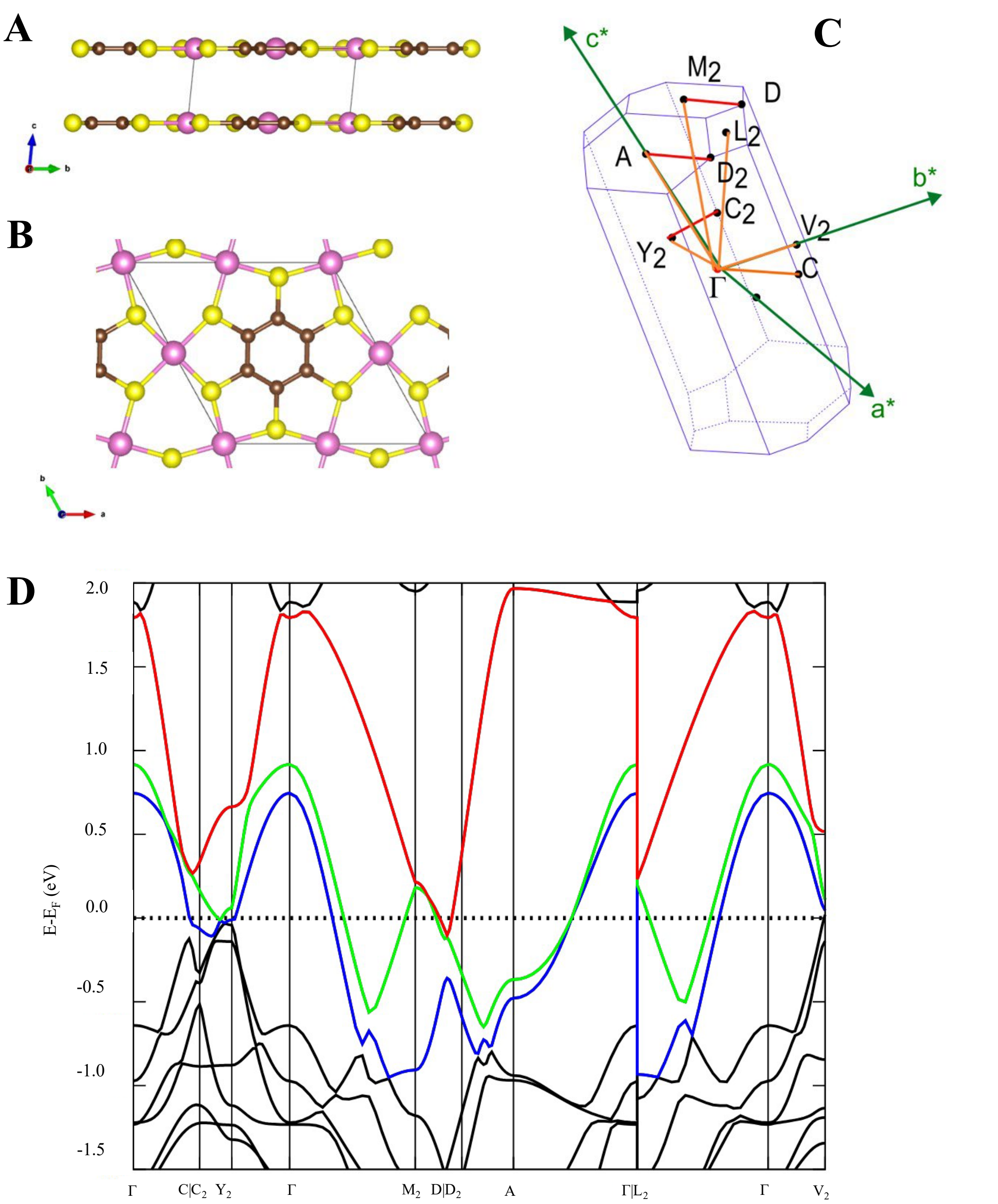

Fig. S21. Relaxed crystal and band structure of CuBHT primitive unit cell in structure "2". This structure *(19)* uses the PBEsol functional from side (**A**) and top (**B**) view. Carbon, sulfur and copper atoms are represented in brown, yellow and pink, respectively; (**C**) Scheme of the primitive brillouin zone with the position of the high symmetry point explored for the band structure calculation; (**D**) Computed band structure at the DFT/PBEsol level of the primitive CuBHT structure. The three bands

crossing the fermi level are represented in blue, red and green. This color code is conserved for the visualization of the fermi surface of the electron and hole pockets (see **Fig. S22-S25**). Results in **Fig. S22-S28** are based on this structure.

Table S3 Comparison of the relaxed parameters using PBE and PBEsol functionals with and without Van der Waals corrections (Grimme D3). The PBEsol without Grimme corrections was used in our calculations as this combination of parameters gives the best results in term of lattice parameters, especially along the interlayer direction (c axis), and preserve the planarity of the CuBHT (see *Δz* parameter defined as the maximum distance along z axis between atoms inside the CuBHT layer).

	PBE	PBE + Grimme D3	PBEsol	PBEsol + Grimme D3
a (Å)	8.767	8.726	8.572	8.639
b (Å)	8.767	8.726	8.572	8.639
c (Å)	3.755	3.429	3.476	3.348
α	99.04	107.64	99.92	108.54
β	99.04	107.64	99.92	108.54
γ	120.03	120.14	120.01	120.06
Δz (Å)	0.06	0.55	0.00	0.55

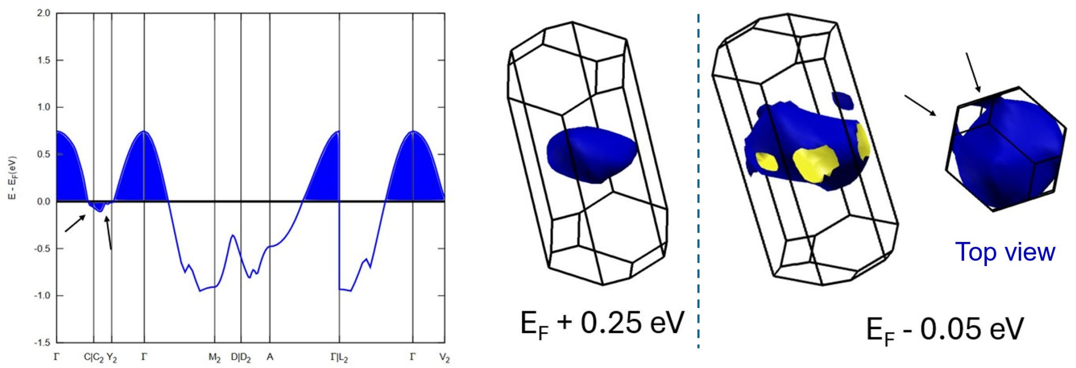

Fig. S22. Electron and hole pockets for the first crossing band. The hole pockets consist in a single fermi surface localized at the Γ point with a quasi-uniform dispersion along the CuBHT plane i.e. (Γ-C, Γ-V_2 or Γ-Y_2 paths) and direction perpendicular to the stacking planes (Γ-A, Γ-L_2). The analysis of the electron pockets shows that the small pockets localized around the C and C_2 k-point are associated to connecting areas between the central Fermi surface (see black arrows).

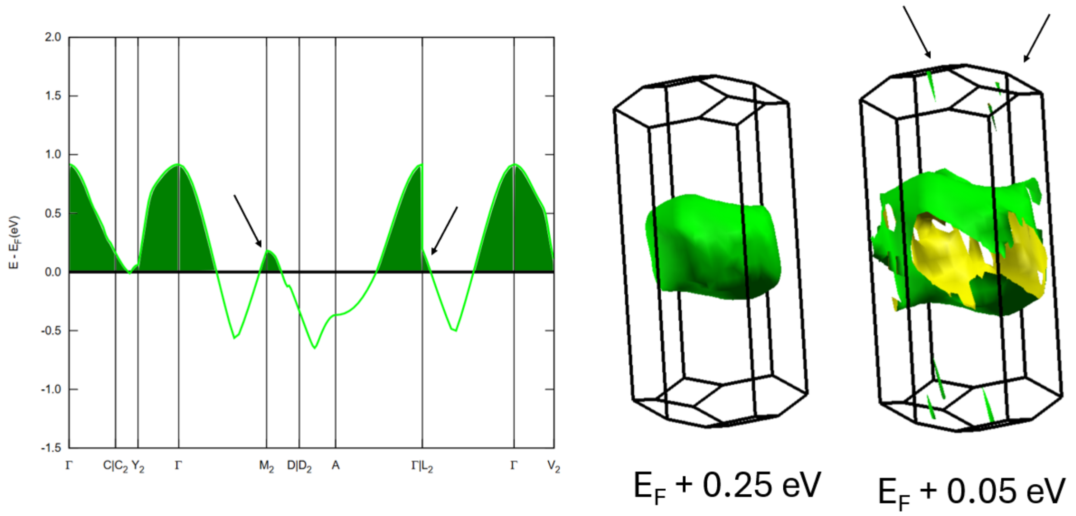

Fig. S23. Hole pockets for the second crossing band. The large pockets correspond to a fermi surface similar to the one already described for the first crossing band. The small pockets are localized at the top of the Brillouin zone at the M_2 (center of a hexagonal facet) and L_2 (center of the square facet) with a dropplet-like shape. However, no pocket is observed at the A point which is the k-point localized along the c* lattice vectors i.e. along the c direction of the structure.

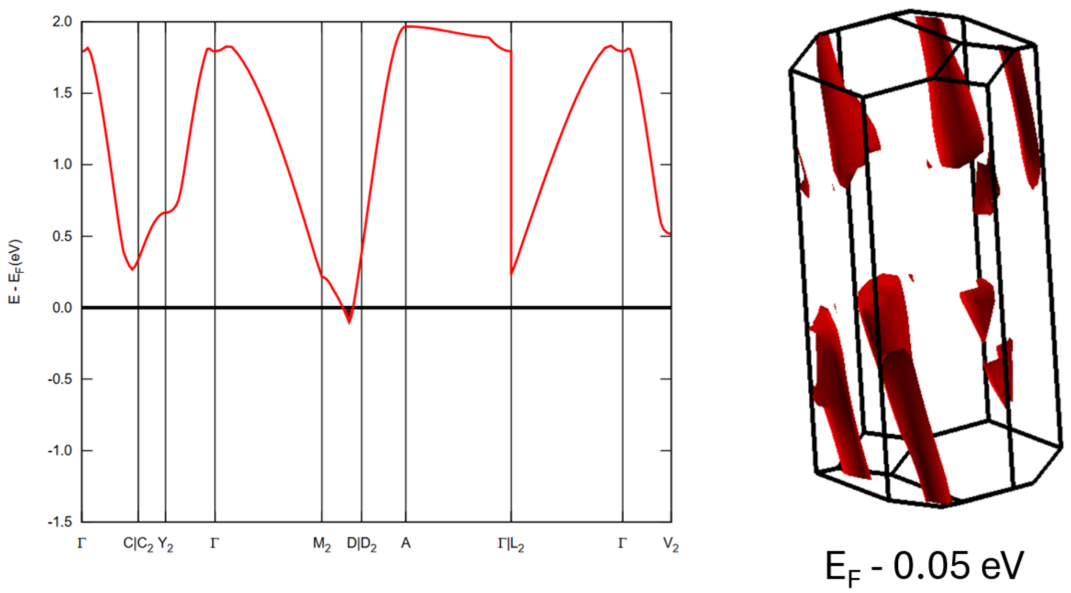

Fig. S24. Electron pockets for the third crossing band. The computed band structure only shows one small pocket between the M_2-D path i.e. at the top of the Brillouin zone. However, the analysis of the full fermi surface just below the fermi level reveals a more complex structure with many tube-like pockets localized at the edge of the Brillouin zone and along the interlayer direction.

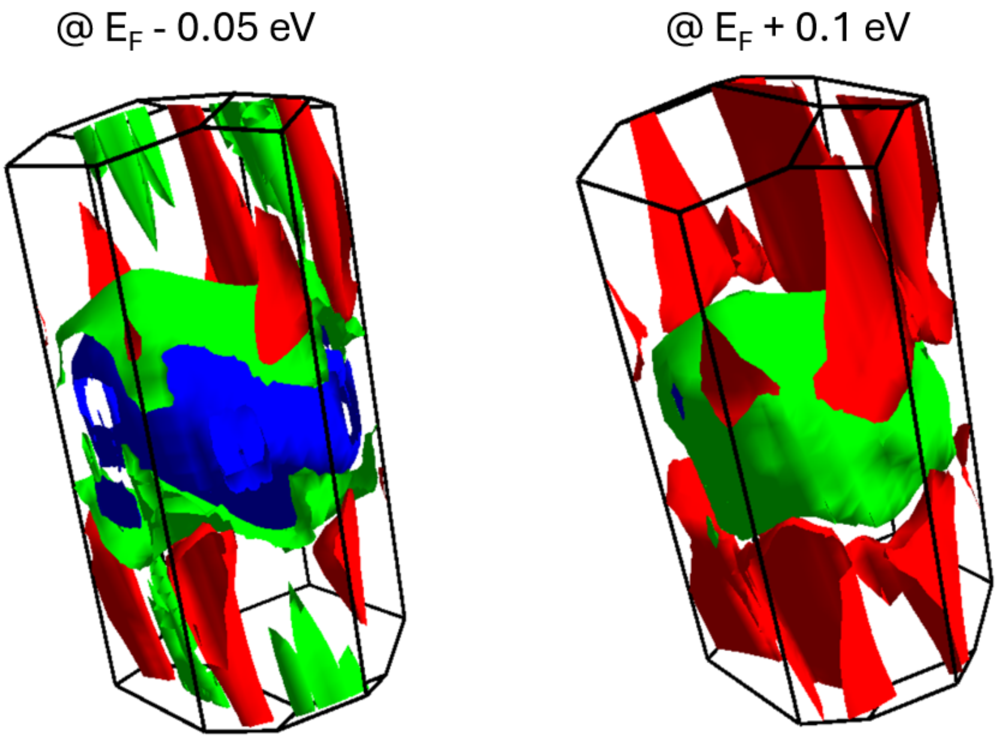

Fig. S25. Fermi surface combining the three crossing bands discussed previously. Energy values were taken at 0.1 eV above the fermi energy (hole pockets) and below 0.05 eV (electron pocket) from the fermi level using the color code described before.

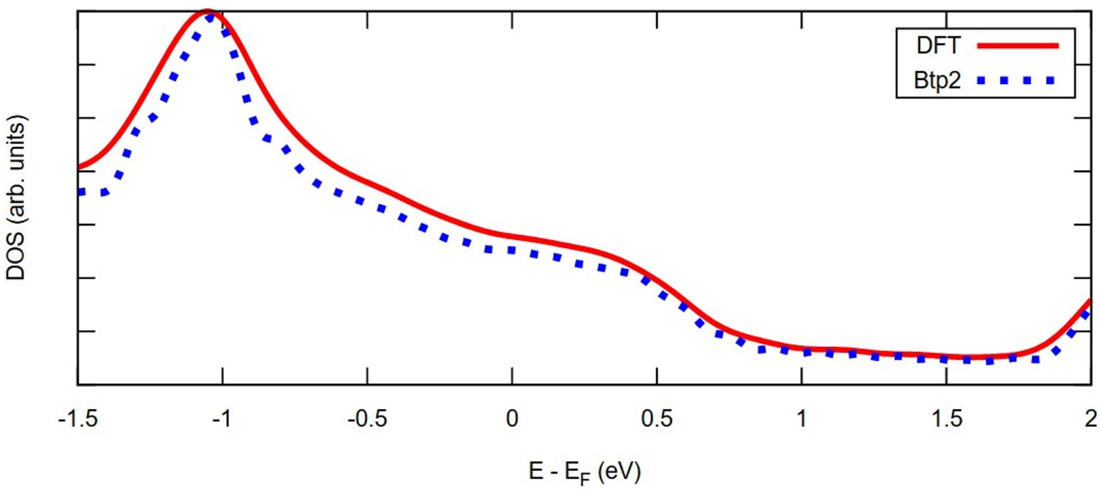

Fig. S26. Energy dependent density of states of CuBHT structure "2". Comparison between the interpolated density of states (DOS) obtained with the BolzTrap2 code (blue dashed line) with the DFT result (red line) around the fermi level based on structure "**2**". The fluctuation of the DOS is nicely reproduced all along the investigated energy window. The calculated DOS also shows symmetric feature around E_F which further confirm near-equal conduction contribution from electrons and holes.

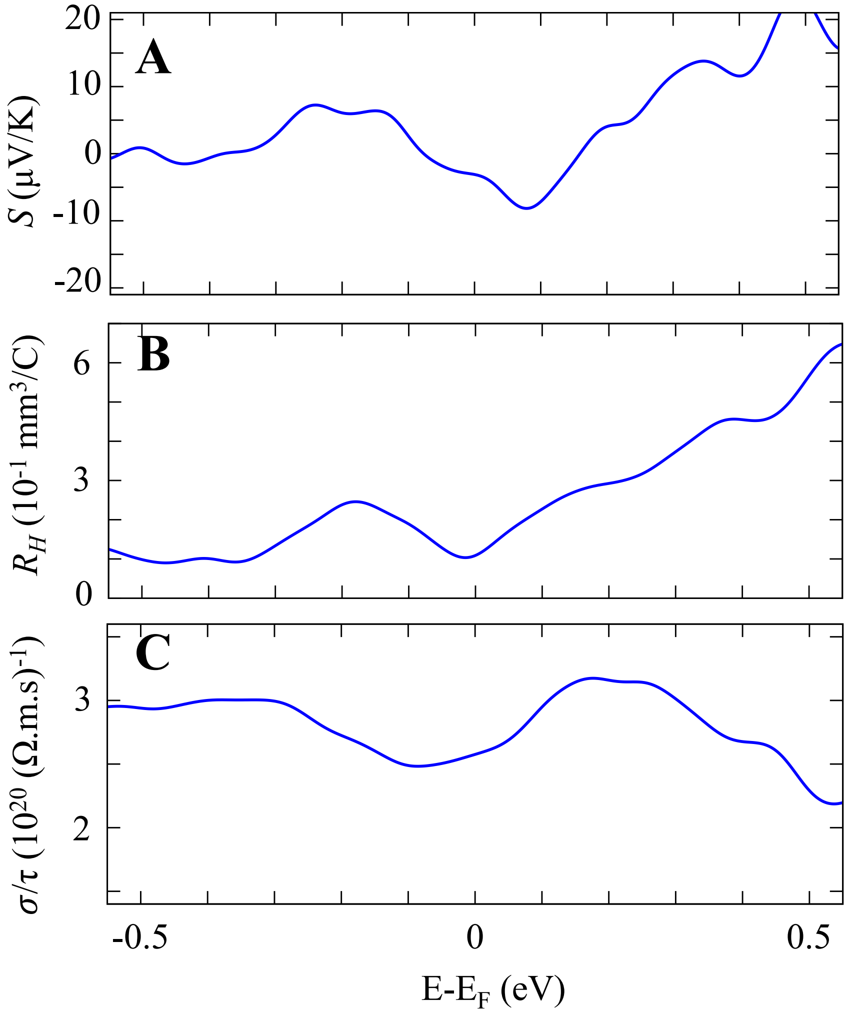

Fig. S27. Computed transport coefficients of CuBHT structure "2". (**A**) Computed Seebeck coefficient in a ± 0.5 eV window around the Fermi level at 300K. A sign inversion is observed at -0.1 eV and +0.15 eV i.e. in the energy window covered by the small electron and hole pockets described before; (**B**) Computed Hall effect in a ± 0.5 eV window around the Fermi level at 300K; (**C**) Computed electronic conductivity divided by the constant relaxation time in a ± 0.5 eV window around the Fermi level at 300K.

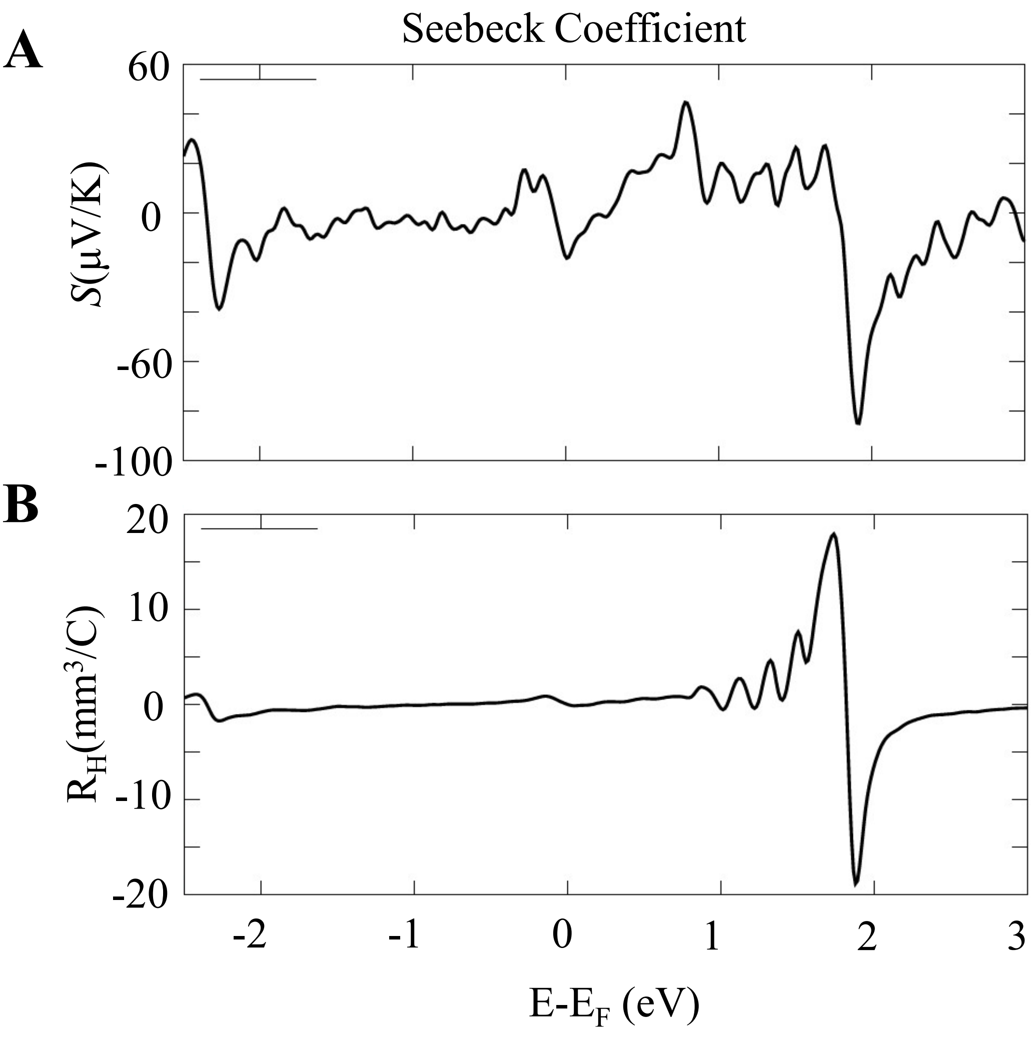

Fig. S28. Computed transport coefficients at wider energy window of another CuBHT structure. (**A**) Computed Seebeck coefficient in a wider energy window (-2.5-3.0 eV) around the Fermi level at 300K; (**B**) Computed Hall effect in a wider window (-2.5-3.0 eV) around the Fermi level at 300K.

REFERENCES AND NOTES

1. K. S. Novoselov, A. K. Geim, S. V. Morozov, D. Jiang, Y. Zhang, S. V. Dubonos, I. V. Grigorieva, A. A. Firsov, Electric field effect in atomically thin carbon films. *Science* **306**, 666–669 (2004).

2. A. K. Geim, Graphene: Status and prospects. *Science* **324**, 1530–1534 (2009).

3. X.-H. Liu, C.-Z. Guan, D. Wang, L.-J. Wan, Graphene-like single-layered covalent organic frameworks: Synthesis strategies and application prospects. *Adv. Mater.* **26**, 6912–6920 (2014).

4. G. Givaja, P. Amo-Ochoa, C. J. Gómez-García, F. Zamora, Electrical conductive coordination polymers. *Chem. Soc. Rev.* **41**, 115–147 (2012).

5. M. D. Allendorf, A. Schwartzberg, V. Stavila, A. A. Talin, A roadmap to implementing metal–organic frameworks in electronic devices: Challenges and critical directions. *Chem. A Eur. J.* **17**, 11372–11388 (2011).

6. R. Sakamoto, K. Takada, T. Pal, H. Maeda, T. Kambe, H. Nishihara, Coordination nanosheets (CONASHs): Strategies, structures and functions. *Chem. Commun.* **53**, 5781–5801 (2017).

7. L. S. Xie, G. Skorupskii, M. Dincă, Electrically conductive metal–organic frameworks. *Chem. Rev.* **120**, 8536–8580 (2020).

8. T. Kusamoto, H. Nishihara, Zero-, one- and two-dimensional bis(dithiolato)metal complexes with unique physical and chemical properties. *Coord. Chem. Rev.* **380**, 419–439 (2019).

9. M. G. Campbell, S. F. Liu, T. M. Swager, M. Dincə, Chemiresistive sensor arrays from conductive 2D metal-organic frameworks. *J. Am. Chem. Soc.* **137**, 13780–13783 (2015).

10. C. H. Hendon, A. J. Rieth, M. D. Korzyński, M. Dincă, Grand challenges and future opportunities for metal-organic frameworks. *ACS Cent. Sci.* **3**, 554–563 (2017).

11. T. Kambe, R. Sakamoto, K. Hoshiko, K. Takada, M. Miyachi, J.-H. Ryu, S. Sasaki, J. Kim, K. Nakazato, M. Takata, H. Nishihara, π-Conjugated nickel bis(dithiolene) complex nanosheet. *J. Am. Chem. Soc.* **135**, 2462–2465 (2013).

12. T. Kambe, R. Sakamoto, T. Kusamoto, T. Pal, N. Fukui, K. Hoshiko, T. Shimojima, Z. Wang, T. Hirahara, K. Ishizaka, S. Hasegawa, F. Liu, H. Nishihara, Redox control and high conductivity of nickel bis(dithiolene) complex π-nanosheet: A potential organic two-dimensional topological insulator. *J. Am. Chem. Soc.* **136**, 14357–14360 (2014).

13. Z. F. Wang, N. Su, F. Liu, Prediction of a two-dimensional organic topological insulator. *Nano Lett.* **13**, 2842–2845 (2013).

14. M. Zhao, A. Wang, X. Zhang, Half-metallicity of a kagome spin lattice: The case of a manganese bis-dithiolene monolayer. *Nanoscale* **5**, 10404–10408 (2013).

15. B. K. Maiti, L. B. Maia, K. Pal, B. Pakhira, T. Avilés, I. Moura, S. R. Pauleta, J. L. Nuñez, A. C. Rizzi, C. D. Brondino, S. Sarkar, J. J. G. Moura, One electron reduced square planar bis(benzene-1,2-dithiolato) copper dianionic complex and redox switch by O_2/HO^-. *Inorg. Chem.* **53**, 12799–12808 (2014).

16. S. S. Staniland, W. Fujita, Y. Umezono, K. Awaga, P. J. Camp, S. J. Clark, N. Robertson, $[BDTA]_2[Cu(mnt)_2]$: An almost perfect one-dimensional magnetic material. *Inorg. Chem.* **44**, 546–551 (2005).

17. X. Huang, P. Sheng, Z. Tu, F. Zhang, J. Wang, H. Geng, Y. Zou, C. A. Di, Y. Yi, Y. Sun, W. Xu, D. Zhu, A two-dimensional π-d conjugated coordination polymer with extremely high electrical conductivity and ambipolar transport behaviour. *Nat. Commun.* **6**, 7408 (2015).

18. X. Zhang, Y. Zhou, B. Cui, M. Zhao, F. Liu, Theoretical discovery of a superconducting two-dimensional metal-organic framework. *Nano Lett.* **17**, 6166–6170 (2017).

19. X. Huang, S. Zhang, L. Liu, L. Yu, G. Chen, W. Xu, D. Zhu, Superconductivity in a copper(II)-based coordination polymer with perfect kagome structure. *Angew. Chem. Int. Ed. Engl.* **130**, 152–156 (2018).

20. T. Takenaka, K. Ishihara, M. Roppongi, Y. Miao, Y. Mizukami, T. Makita, J. Tsurumi, S. Watanabe, J. Takeya, M. Yamashita, K. Torizuka, Y. Uwatoko, T. Sasaki, X. Huang, W. Xu, D. Zhu, N. Su, J.-G. Cheng, T. Shibauchi, K. Hashimoto, Strongly correlated superconductivity in a copper-based metal-organic framework with a perfect kagome lattice. *Sci. Adv.* **7**, eabf3996 (2021).

21. X. Huang, S. Fu, C. Lin, Y. Lu, M. Wang, P. Zhang, C. Huang, Z. Li, Z. Liao, Y. Zou, J. Li, S. Zhou, M. Helm, P. St. Petkov, T. Heine, M. Bonn, H. I. Wang, X. Feng, R. Dong, Semiconducting conjugated coordination polymer with high charge mobility enabled by “4 + 2” phenyl ligands. *J. Am. Chem. Soc.* **145**, 2430–2438 (2023).

22. R. Tsuchikawa, N. Lotfizadeh, N. Lahiri, S. Liu, M. Lach, C. Slam, J. Louie, V. V. Deshpande, Unique thermoelectric properties induced by intrinsic nanostructuring in a polycrystalline thin-film two-dimensional metal–organic framework, copper benzenehexathiol. *Phys. Status Solidi A Appl. Matter Sci.* **217**, 2000437 (2020).

23. R. Toyoda, N. Fukui, D. H. L. Tjhe, E. Selezneva, H. Maeda, C. Bourgès, C. M. Tan, K. Takada, Y. Sun, I. Jacobs, K. Kamiya, H. Masunaga, T. Mori, S. Sasaki, H. Sirringhaus, H. Nishihara, Heterometallic benzenehexathiolato coordination nanosheets: Periodic structure improves crystallinity and electrical conductivity. *Adv. Mater.* **34**, 2106204 (2022).

24. N. Xin, J. Lourembam, P. Kumaravadivel, A. E. Kazantsev, Z. Wu, C. Mullan, J. Barrier, A. A. Geim, I. V Grigorieva, A. Mishchenko, A. Principi, V. I. Fal’ko, L. A. Ponomarenko, A. K. Geim, A. I. Berdyugin, Giant magnetoresistance of Dirac plasma in high-mobility graphene. *Nature* **616**, 270–274 (2023).

25. A. H. Mayo, H. Takahashi, S. Ishiwata, K. Górnicka, M. J. Winiarski, J. Jaroszynski, R. J. Cava, W. Xie, T. Klimczuk, Enhancement of the magnetoresistance in the mobility-engineered compensated metal Pt_5P_2. *Adv. Electron. Mater.* **9**, 2201120 (2023).

26. K. Kang, S. Watanabe, K. Broch, A. Sepe, A. Brown, I. Nasrallah, M. Nikolka, Z. Fei, M. Heeney, D. Matsumoto, K. Marumoto, H. Tanaka, S.-I. Kuroda, H. Sirringhaus, 2D coherent

charge transport in highly ordered conducting polymers doped by solid state diffusion. *Nat. Mater.* **15**, 896–902 (2016).

27. D. Venkateshvaran, A. J. Kronemeijer, J. Moriarty, D. Emin, H. Sirringhaus, Field-effect modulated Seebeck coefficient measurements in an organic polymer using a microfabricated on-chip architecture. *APL Mater.* **2**, 032102 (2014).

28. M. Statz, S. Schneider, F. J. Berger, L. Lai, W. A. Wood, M. Abdi-Jalebi, S. Leingang, H.-J. Himmel, J. Zaumseil, H. Sirringhaus, Charge and thermoelectric transport in polymer-sorted semiconducting single-walled carbon nanotube networks. *ACS Nano* **14**, 15552–15565 (2020).

29. S. J. Mason, A. Hojem, D. J. Wesenberg, A. D. Avery, B. L. Zink, Determining absolute Seebeck coefficients from relative thermopower measurements of thin films and nanostructures. *J. Appl. Phys.* **127**, 085101 (2020).

30. E. H. Sondheimer, The theory of the galvanomagnetic and thermomagnetic effects in metals. *Proc. R. Soc. London Ser. A. Math. Phys. Sci.* **193**, 484–512 (1948).

31. R. Bel, K. Behnia, H. Berger, Ambipolar Nernst effect in $NbSe_2$. *Phys. Rev. Lett.* **91**, 066602 (2003).

32. X. Zhou, H. Liu, W. Wu, K. Jiang, Y. Shi, Z. Li, Y. Sui, J. Hu, J. Luo, Anomalous thermal Hall effect and anomalous Nernst effect of CsV_3Sb_5. *Phys. Rev. B* **105**, 205104 (2022).

33. A. B. Pippard, *Magnetoresistance in Metals* (Cambridge Univ. Press, 1989).

34. C.-Z. Li, J.-G. Li, L.-X. Wang, L. Zhang, J.-M. Zhang, D. Yu, Z.-M. Liao, Two-carrier transport induced Hall anomaly and large tunable magnetoresistance in Dirac semimetal Cd_3As_2 nanoplates. *ACS Nano* **10**, 6020–6028 (2016).

35. X. Huang, L. Zhao, Y. Long, P. Wang, D. Chen, Z. Yang, H. Liang, M. Xue, H. Weng, Z. Fang, X. Dai, G. Chen, Observation of the chiral-anomaly-induced negative magnetoresistance in 3D Weyl semimetal TaAs. *Phys. Rev. X* **5**, 031023 (2015).

36. K. Durczewski, M. Ausloos, Theory of the thermoelectric power or Seebeck coefficient: The case of phonon scattering for a degenerate free-electron gas. *Phys. Rev. B* **53**, 1762–1772 (1996).

37. Y. Wang, Y.-J. Hu, B. Bocklund, S.-L. Shang, B.-C. Zhou, Z.-K. Liu, L.-Q. Chen, First-principles thermodynamic theory of Seebeck coefficients. *Phys. Rev. B* **98**, 224101 (2018).

38. Z. Pan, X. Huang, Y. Fan, S. Wang, Y. Liu, X. Cong, T. Zhang, S. Qi, Y. Xing, Y.-Q. Zheng, J. Li, X. Zhang, W. Xu, L. Sun, J. Wang, J.-H. Dou, Synthesis and structure of a non-van-der-Waals two-dimensional coordination polymer with superconductivity. *Nat. Commun.* **15**, 9342 (2024).

39. H. Hu, M. P. Singh, G. S. Baghel, G. W. Dye, D. L. Gerlach, T. P. Vaid, Synthesis of 9,10-dimethyl-2,3,6,7-anthracenetetra(thioacetate) and benzenepentathiol; improved syntheses of 1,2,4,5-benzenetetra(thioacetate) and benzenehexathiol. *ChemistrySelect* **1**, 2163–2166 (2016).

40. G. Kresse, J. Hafner, Ab initio molecular dynamics for liquid metals. *Phys. Rev. B* **47**, 558–561 (1993).

41. G. Kresse, D. Joubert, From ultrasoft pseudopotentials to the projector augmented-wave method. *Phys. Rev. B* **59**, 1758–1775 (1999).

42. J. P. Perdew, A. Ruzsinszky, G. I. Csonka, O. A. Vydrov, G. E. Scuseria, L. A. Constantin, X. Zhou, K. Burke, Restoring the density-gradient expansion for exchange in solids and surfaces. *Phys. Rev. Lett.* **100**, 136406 (2008).

43. Y. Hinuma, G. Pizzi, Y. Kumagai, F. Oba, I. Tanaka, Band structure diagram paths based on crystallography. *Comput. Mater. Sci.* **128**, 140–184 (2017).

44. A. Kokalj, Computer graphics and graphical user interfaces as tools in simulations of matter at the atomic scale. *Comput. Mater. Sci.* **28**, 155–168 (2003).

45. M. Kawamura, FermiSurfer: Fermi-surface viewer providing multiple representation schemes. *Comput. Phys. Commun.* **239**, 197–203 (2019).

46. G. K. H. Madsen, J. Carrete, M. J. Verstraete, BoltzTraP2, a program for interpolating band structures and calculating semi-classical transport coefficients. *Comput. Phys. Commun.* **231**, 140–145 (2018).

47. S. Hikami, A. I. Larkin, Y. Nagaoka, Spin-orbit interaction and magnetoresistance in the two dimensional random system. *Prog. Theor. Phys.* **63**, 707–710 (1980).

48. W. A. Wood, T. Marsh, H. Sirringhaus, JISA: A Polymorphic Test-and-Measurement Automation Library. arXiv:2308.13127 [physics.ins-det] (2023).
